# Altered intestinal microbiota–host mitochondria crosstalk in new onset Crohn's disease

**DOI:** 10.1038/ncomms13419

**Published:** 2016-11-23

**Authors:** Walid Mottawea, Cheng-Kang Chiang, Marcus Mühlbauer, Amanda E. Starr, James Butcher, Turki Abujamel, Shelley A. Deeke, Annette Brandel, Hu Zhou, Shadi Shokralla, Mehrdad Hajibabaei, Ruth Singleton, Eric I. Benchimol, Christian Jobin, David R. Mack, Daniel Figeys, Alain Stintzi

**Affiliations:** 1 Department of Biochemistry, Microbiology and Immunology, University of Ottawa, 451 Smyth Road, Ottawa, Ontario, Canada K1H 8M5; 2 Ottawa Institute of Systems Biology, University of Ottawa, 451 Smyth Road, Ottawa, Ontario, Canada K1H 8M5; 3 Department of Microbiology and Immunology, Faculty of Pharmacy, Mansoura University, Mansoura 35516, Egypt; 4 Department of Medicine, Division of Gastroenterology, Hepatology and Nutrition, University of Florida, Gainesville, Florida 32611, USA; 5 Shanghai Institute of Materia Medica, Chinese Academy of Sciences, Shanghai 201203, China; 6 Biodiversity Institute of Ontario, Department of Integrative Biology, University of Guelph, Guelph, Ontario, Canada N1G 2W1; 7 Children's Hospital of Eastern Ontario (CHEO) Inflammatory Bowel Disease Centre and CHEO Research Institute, 451 Smyth Road, Ottawa, Ontario, Canada K1H 8L1; 8 Department of Pediatrics, University of Ottawa, 451 Smyth Road, Ottawa, Ontario, Canada K1H 8M5; 9 School of Epidemiology, Public Health and Preventive Medicine, University of Ottawa, 451 Smyth Road, Ottawa, Ontario, Canada K1H 8M5; 10 Department of Medicine, Department of Infectious Diseases and Pathology, University of Florida, Gainesville, Florida 32611, USA; 11 Department of Chemistry and Biomolecular Sciences, University of Ottawa, 451 Smyth Road, Ottawa, Ontario, Canada K1H 8M5

## Abstract

Intestinal microbial dysbiosis is associated with Crohn's disease (CD). However, the mechanisms leading to the chronic mucosal inflammation that characterizes this disease remain unclear. In this report, we use systems-level approaches to study the interactions between the gut microbiota and host in new-onset paediatric patients to evaluate causality and mechanisms of disease. We report an altered host proteome in CD patients indicative of impaired mitochondrial functions. In particular, mitochondrial proteins implicated in H_2_S detoxification are downregulated, while the relative abundance of H_2_S microbial producers is increased. Network correlation analysis reveals that *Atopobium parvulum* controls the central hub of H_2_S producers. *A. parvulum* induces pancolitis in colitis-susceptible interleukin-10-deficient mice and this phenotype requires the presence of the intestinal microbiota. Administrating the H_2_S scavenger bismuth mitigates *A. parvulum*-induced colitis *in vivo*. This study reveals that host–microbiota interactions are disturbed in CD and thus provides mechanistic insights into CD pathogenesis.

## Abstract (web summary)

Crohn's disease is associated with altered intestinal microbiota. Here, the authors show that the microbe *Atopobium parvulum* is associated with Crohn's disease patients, triggers colitis in a mouse model, and that scavenging microbe-induced hydrogen sulfide improved symptoms in mice.

## Graphical abstract

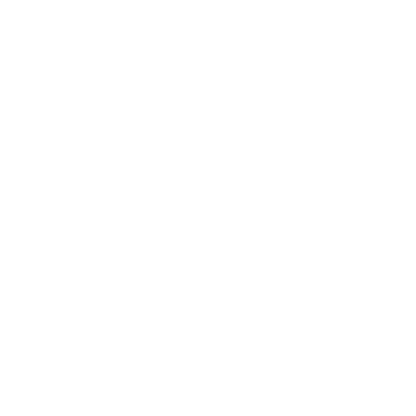

Inflammatory bowel disease (IBD) is characterized by chronic and relapsing mucosal inflammation of the gastrointestinal tract and comprises two main subtypes, Crohn's disease (CD) and ulcerative colitis (UC)^1^. A prevailing hypothesis is that IBD development is a consequence of aberrant interplay between an altered intestinal microbiota with the host^2^. The composition of the intestinal microbiota from IBD patients is typically characterized by decreased prevalence of protective microorganisms (that is, Clostridium XIVa and IV groups) and an expansion of detrimental bacteria (that is, Enterobacteriaceae and Fusobacteria)^2^,^3^,^4^,^5^,^6^,^7^. However, there is limited information on causality and mechanism of disease. Moreover, a fundamental unanswered question is whether the observed microbial dysbiosis is a cause or a consequence of inflammation.

To address causality and microbial dysbiosis in CD pathogenesis, we conducted a systems-level study of the interaction between the intestinal microbiota and host at the mucosa–luminal interface (MLI) in newly diagnosed paediatric patients. New-onset, paediatric subjects constitute an important population to study the role of the intestinal microbiota in IBD as there are no treatment effects on the disease process, co-morbidities are rare and there are fewer confounders as compared with adults. In addition, the inflammatory phase of IBD characteristically predominates in younger subjects and thus, these patients are more likely to reveal the underlying mechanisms that promote early development of disease. To further identify key microbial drivers of inflammation, we characterized the MLI microbial community at the time of diagnostic endoscopy. In contrast to stool microbiota, the MLI microbial community is in direct contact with the site of inflammation and is therefore likely to be directly involved in the initiation and maintenance of the diseased state. To understand the interactions between the intestinal microbiome and the host, we characterized the host proteome and identified key associations between the host proteome and intestinal microbiota. We also validated the causative role of a microbe that predicts disease severity in a mouse model of colitis. The use of these approaches in the context of a phenotypically well-defined patient cohort has enabled us to elucidate a possible mechanism of disease and to identify key microbial drivers of inflammation.

## Results

### Characteristics of the paediatric IBD gut microbiota

**Figure 1:**
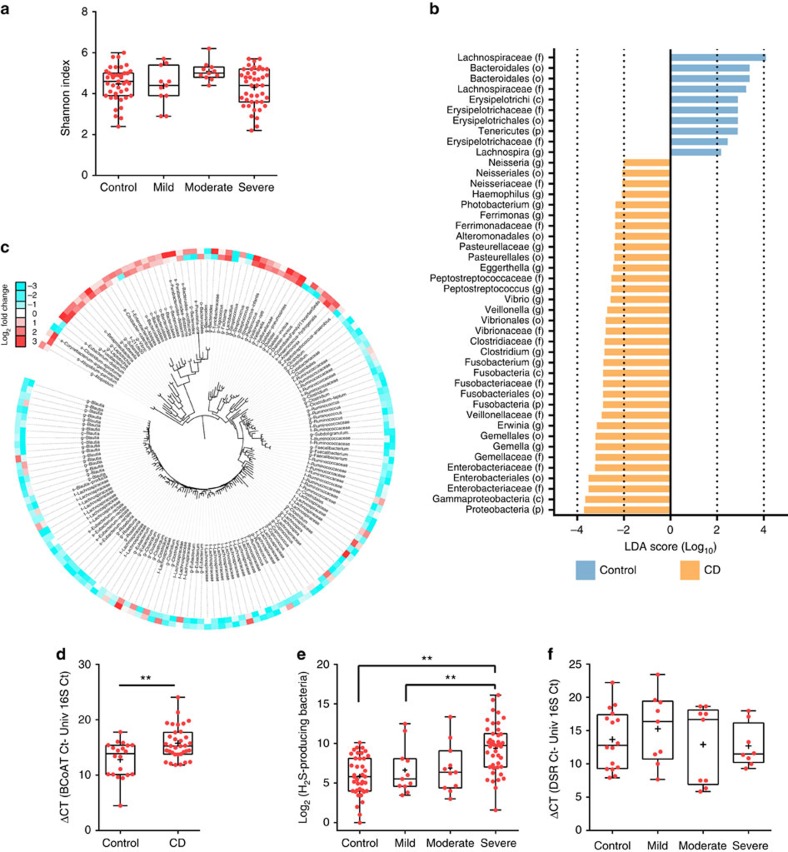
Assessment of the microbiota composition at the mucosa-luminal interface of new-onset paediatric IBD. (**a**) Diversity of the intestinal microbiota in control and CD paediatric patients as a function of disease severity. Shannon index was calculated using 500,000 reads per sample. Crosses indicate the mean while horizontal lines indicate the median. (**b**) Histogram of linear discriminant analysis (LDA) effect size score for CD differentially abundant taxa compared with controls (*n*=65 and 42 for CD and controls, respectively); only OTUs meeting an LDA significant threshold ≥2 with a *P*<0.05 (pairwise Wilcoxon test) are shown and are denoted with their lowest defined taxonomy. (**c**) Phylogenetic tree of the differentially abundant OTUs identified by metagenomeSeq analysis (fold change ≥2 and *P*<0.05); an increasing red intensity indicates OTUs whose relative abundance increased, whereas an increasing blue intensity indicates OTUs whose relative abundance decreased in CD patients with severe inflammation as compared with mild (outer circle) or severe as compared with moderate (inner circle); the maximum colour output for this figure was set at a Log_2_ value of ±3. (**d**) The qPCR quantification of butyrate producing bacteria in control and CD microbiota. Butyryl-CoA: acetyl-CoA transferase gene (BCoAT); *n*=20 and 34 for control and CD, respectively; statistical comparison by Mann–Whitney two-tailed test; **P<0.01). (**e**) Log_2_ sum of rarefied reads assigned to taxa known to produce H_2_S through amino acid fermentation were plotted for the control and as a function of CD severity (*n*=39, 11, 11, 43 for control subjects and mild, moderate and severe CD, respectively). (**f**) The qPCR quantification of sulfate reducing bacteria in control and CD patient microbiota as a function of disease severity. Dissimilatory sulfide reductase gene (DSR); *n*=16, 9, 9 and 8 for control, mild, moderate and severe CD, respectively). (**a**,**e**,**f**) Statistical comparison by Kruskal–Wallis test using Dunn's *post hoc* test and followed by a Bonferroni correction for the significance level; *P<0.05; **P<0.01. Crosses indicate the mean while horizontal lines indicate the median.

Although others have previously characterized the microbiota composition at the mucosa-luminal interface (MLI) in adult cohorts^8^,^9^, the MLI of new-onset paediatric IBD patients has not been previously reported. Given the intimate location of the MLI microbiota on the host intestinal epithelium, we hypothesized that studying the microbial composition at this location would provide direct information on how microbes affect inflammation and gut physiological processes. To characterize the composition of the MLI microbiota in IBD, we collected the MLI from the ascending colon of new-onset paediatric IBD patients and controls by flushing sterile water onto the mucosa to dislodge the mucus layer from the mucosal epithelial cells followed by aspiration of these mucosal lavages through the colonoscope (Table 1 and Supplementary Table 1). We investigated the MLI microbiota using 16S rDNA-based Illumina sequencing on a total of 65 CD, 21 UC patients and 42 control subjects with the remaining samples from our cohort (29 CD, 16 UC and 21 controls) used for independent validation with alternative quantification approaches (that is, Ion Torrent/454 sequencing and quantitative PCR (qPCR)). Microbial diversity was unchanged between controls and the CD/UC patients (Fig. 1a and Supplementary Figs 1 and 2) in contrast to the current model of decreased diversity in IBD patients^10^, but in agreement with a previous study on new-onset paediatric IBD patients^3^. These results suggest that first-onset paediatric patients have a distinct microbial profile as compared with adults and post-treatment cohorts.

Phylotypes differentially abundant between CD and controls or UC and controls were identified using a linear discriminant analysis effect size method^11^ and a zero-inflated Gaussian mixture model (metagenomeSeq)^12^. These two statistical methods identified most of the same key microbes (Fig. 1b and Supplementary Fig. 3; and Supplementary Data 1 and 2). In addition, to ensure that our use of the V6 hypervariable region as opposed to the V4 region commonly used in microbiota studies did not unduly influence our results, we sequenced the V4 and V6 regions in a subset of our samples using Ion Torrent sequencing. Importantly, while there are obviously unique taxa identified as being differentially abundant using each approach (Supplementary Fig. 4, Supplementary Data 3), many of the same key taxa are identified using each technique. Moreover, these results are highly concordant with those obtained by Gevers *et al*.^3^, who characterized the microbiota composition of new-onset paediatric CD patients using rectal mucosal biopsies instead of MLI samples. These include increased Clostridiales and Bacteroidales in controls and increased Enterobacteriaceae, *Veillonella*, *Fusobacterium*, *Neisseria* and *Haemophilus* in CD patients (Supplementary Data 2). The identification of the same key players highlights the usefulness of MLI as biological samples and the high quality of our inception cohort.

Numerous reports have cautioned that 16S rDNA results can be confounded by the presence of sequencing artifacts and/or contamination from the kits used during 16S library construction^13^. To quantify the levels of these artifacts and contaminants in our experimental setup, we purified genomic DNA from pure bacterial cultures and subsequently processed and analysed these samples as done for our MLI aspirates. These experiments revealed that our sequencing approach yields accurate results, with each pure bacterial sample primarily composed of a single dominant operational taxonomic unit (OTU) matching the genus of the sequenced bacteria with a median relative abundance of ∼99.1% (Supplementary Fig. 5). Using this data set, we also determined that our median PCR/sequencing error rate was 0.007, which is comparable to those obtained in other studies^14^ (Supplementary Fig. 6). In addition, we determined that potential contamination from our sequencing reagents/kits accounted for a median of ∼0.6% relative abundance. Importantly, the levels of sequencing artifacts and contamination quantified in these control experiments are low enough to be confidently removed during our bioinformatic processing and statistical analyses (‘Methods' section).

### Key phylotypes correlating with CD inflammation severity

To identify candidate causal microbes of CD pathogenesis, we examined changes in colonic microbiota composition at different stages of the disease from mild to moderate to severe inflammation (as determined by the PCDAI (Pediatric Crohn's Disease Activity Index) score). As taxonomy binning might mask important microbial dynamic behaviour, we identified key phylotypes correlating with CD inflammation severity at the OTU level (defined by sequence clustering at 97% identity as a proxy of species-level resolution). Non-supervised multidimensional scaling analysis failed to separate the microbial communities as a function of disease severity likely due to high interpersonal microbial variation (Supplementary Fig. 7) and instead segregated by microbial diversity as reported by others^3^. Nevertheless, we assessed the association of individual OTUs with disease severity using generalized linear models and controlling for potential confounders (gender and inflammation status of the sampled site). A total of 161 OTUs were found to strongly correlate, (53 OTUs positively and 108 OTUs negatively), with disease severity (Fig. 1c; Supplementary Data 4). The OTUs negatively correlating with severity include major butyrate producers such as *Blautia*, Lachnospiraceae, *Roseburia*, *Eubacterium rectale*, *Ruminococcus*, *Clostridium* and *Faecalibacterium*. This decrease in butyrate producers was further confirmed using a qPCR assay targeting the butyryl-CoA:acetate CoA-transferase (BCoAT) gene, which encodes a key enzyme of the major pathway for butyrate production in the gut environment. As expected, CD patients had significantly fewer copies of the BCoAT gene as compared with the control subjects indicating a decreased microbial capacity to produce butyrate (Fig. 1d). The OTUs found to positively correlate with severity include members of the order Bacteriodales, the families Enterobacteriaceae and Veillonellaceae, and the genera *Atopobium*, *Fusobacterium*, *Leptotrichia*, *Sutterella*, *Vibrio*, *Parabacteroides*, *Prevotella*, *Peptostreptococcus*, *Peptococcus* and *Streptococcus*. It is noteworthy that one-fourth of these OTUs (*Atopobium*, *Fusobacterium*, *Veillonella*, *Prevotella*, *Streptoccocus* and *Leptotrichia*) are known to produce H_2_S through the fermentation of sulfur-containing amino-acids^15^. We therefore evaluated whether the relative abundance of these H_2_S producers could predict CD severity using receiver-operating characteristic analysis. These H_2_S producers predicted severe inflammation with an AUC of 0.74 (95% confidence intervals 0.61 to 0.87; Supplementary Fig. 8) indicating that this group of microbes could be used to classify CD patients with severe inflammation. Indeed, the relative abundance of these H_2_S producers correlated positively with the severity of inflammation (Fig. 1e). H_2_S can also be produced by sulfate-reducing bacteria (SRB) but we found no evidence of a link between the SRB and CD patients as shown by a qPCR assay targeting the dissimilatory sulfite reductase gene (Fig. 1f). All together, these results suggest a possible role for the H_2_S-producing bacteria in CD pathogenesis through the fermentation of sulfur-containing compounds and not through dissimilatory sulfate reduction.

### *Atopobium parvulum* is a key network hub

**Figure 2:**
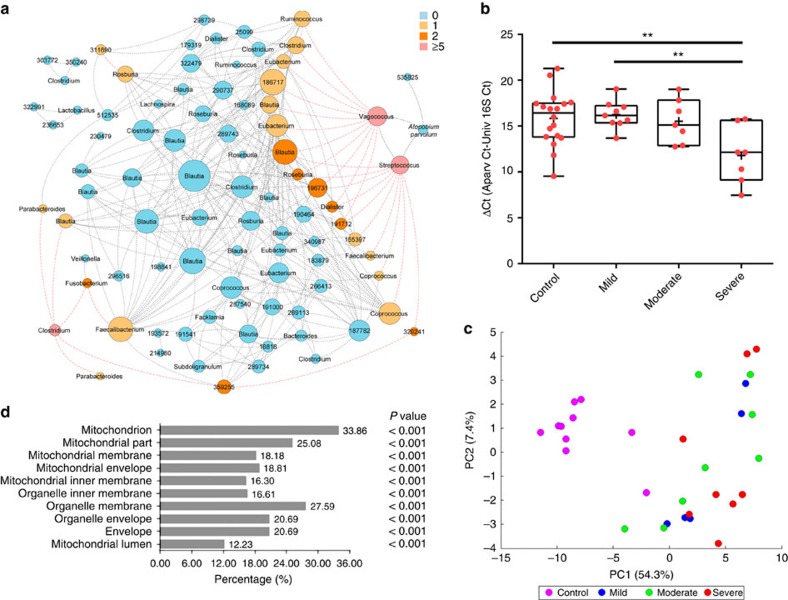
Enrichment of *A. parvulum* and altered mitochondrial proteome define the severity of Crohn's disease. (**a**) Interaction network of differentially abundant OTUs; each node represents an OTU and are sized according to their number of interactions; each edge denotes a significant co-exclusion (red) or co-occurence (grey) relationship between OTUs. Nodes are coloured by their number of significant co-exclusions. (**b**) The qPCR quantification of *A. parvulum* in control and CD microbiota as a function of disease severity. ΔCt was calculated by subtracting average Ct values of universal 16S rDNA from average Ct values of *A. parvulum* specific primers (*n*=18 for controls, nine for mild CD and seven for moderate and severe CD). **P<0.01 estimated using Kruskal–Wallis followed by Dunn's *post hoc* test. Crosses indicate the mean while horizontal lines indicate the median. (**c**) Principal component analysis of the differentially expressed proteins among controls and CD patients categorized as a function of disease severity. (**d**) Functional annotation (cellular component) analysis of the differentially expressed proteins; the 10 most significantly enriched functional groups (GO terms) are shown (Fisher's exact test *P*<10^*−*13^). The illustrated *P* values are for classifications that were significantly enriched compared to the whole proteomic data set.

To predict interactions among individual OTUs, we constructed a network of correlations between the abundance of the OTUs found to be associated with disease severity (Fig. 2a). The resulting network included 89 nodes (OTUs) and 341 edges (representing 312 co-occurrence and 29 co-exclusion interactions). Two major co-excluded modules of OTUs appeared in the network analysis separating OTUs on the basis of their relative abundance with respect to inflammation (increased or decreased in CD as compared with control subjects). One module of co-occurring OTUs consisted of *Vagoccocus* (OTU-570199), *Streptococcus* (OTU-218959), OTU-535825 (Veillonellaceae) with *A. parvulum* (OTU-529659) as the central hub of this module. The OTUs from this module were all found to be increased in CD. The *A. parvulum*-module anti-correlated with the other major module, which is mostly composed of OTUs that are decreased in CD and belong to the class Clostridia. The relative abundance of *A. parvulum* was validated by qPCR and found to be positively correlated with CD severity (Fig. 2b). The correlation between *A. parvulum* and CD was also confirmed by 454 pyrosequencing of a subset of samples followed by linear discriminant analysis effect size method analysis (Supplementary Fig. 9).

### Functional alterations in the paediatric CD host proteome

To gain mechanistic insights into the role of microbes in CD severity, we conducted an unbiased, shotgun, quantitative proteomic analysis of mucosal biopsies from controls (*n*=10) and CD (*n*=21) subjects with various levels of disease severity and tested for associations between these host–proteomes and the intestinal microbes. A total 3,323 proteins were quantified using the superSILAC proteomics approach described. The majority of proteins were quantifiable within a 10-fold tissue/super-SILAC ratio (85.7%: non-normalized L/H ratios, 89.3%: normalized L/H ratios) in all the samples (Supplementary Fig. 10a). Within the 475 proteins quantified by ratios outside the range of 10-fold in at least one sample (non-normalized L/H ratios), only 91 proteins had a median ratio above 10-fold (Supplementary Fig. 10b). This is comparable to a recently published superSILAC proteome study^16^ that showed that 83.7% of identified proteins were within a 10-fold ratio. There were 39 proteins identified in the light samples that did not have a heavy counterpart (Supplementary Fig. 10c). Of these, only one protein (Ribonuclease pancreatic; Uniprot ID: P07998) was detected in more than half of the samples without a corresponding SILAC-labelled peak from super-SILAC standard. Taken together, these results highlight the applicability of the reference proteome for use with the human biopsy sample proteomes.

Three hundred and twenty of the 3,323 quantified proteins were identified as differentially expressed by comparing the CD patients versus control (*t*-test with *q*<0.05). PCA of the proteomic profiles grouped the control subjects away from the CD patients with the first two components explaining 61.7% of the variation (Fig. 2c). KEGG pathway analysis, through the DAVID bioinformatics resources, revealed the top 10 enriched pathways (Fisher's exact test *P*<0.01; Supplementary Fig. 11). Gene ontology analysis identified the mitochondrial proteins as the major discriminant feature of all differentially expressed proteins (Fig. 2d). Most mitochondrial proteins (93.5%; 100/107 mitochondrial proteins) were found to be significantly downregulated in the CD patients as compared with controls (Fisher's exact test *P*<0.05) with the extent of downregulation rising with increased severity (Supplementary Fig. 12). Interestingly, components of the mitochondrial hydrogen sulfide detoxification complex^17^, namely the sulfur dioxygenase (*ETHE1*), the thiosulfate sulfurtransferase (*TST*) and the components of complexes III and IV of the mitochondrial respiratory chain, were downregulated in the CD patients compared with controls (two-tailed Mann–Whitney test *P*<0.05). Secondary validation by qRT–PCR confirmed the repression of *tst*, cytochrome *c* oxidase subunit IV (*hcox41*) and the sulfide dehydrogenase genes (*SQRDL*) transcripts, all of which detoxify H_2_S, in CD and/or UC patients (Supplementary Fig. 13). These findings indicate that the decreased abundance of these H_2_S-detoxification proteins is a hallmark of CD disease activity and possibly UC as well.

**Figure 3:**
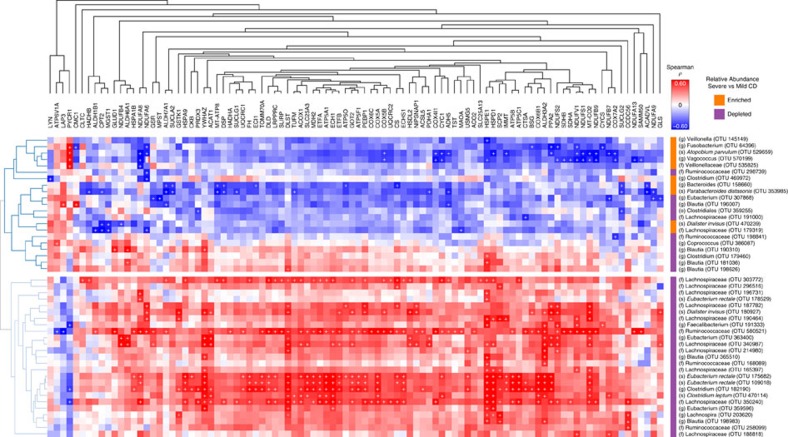
Microbiota–mitochondria correlation analysis. Clustered heatmap of Spearman–Kendall correlation analysis between differentially abundant OTUs and mitochondrial proteins; red and blue colours indicate a positive or negative Spearman correlation respectively (*P* value <0.05 for both the Kendall Tau and the Spearman's rank statistics was used to define significance denoted by ‘+'). Note: only OTUs and mitochondrial proteins with at least one significant correlation are shown. Whether the OTU was increased or decreased in mild versus severe CD patients is denoted on the bar to the left of the OTU name (blue decreased, red increased).

We next performed ‘transkingdom' correlation analysis between the levels of differentially expressed mitochondrial proteins and the OTUs that correlated with CD inflammation severity (Fig. 3). In total, 46 OTUs displayed significant associations with 96 host mitochondrial proteins with a *P*<0.05 in using both Kendall and Spearman correlations. Interestingly, OTUs that were found to be decreased in CD displayed strong positive correlations with mitochondrial proteins while OTUs found to be increased mainly displayed negative correlations. Of particular note, OTUs displaying positive correlations are known butyrate producers. OTU-580521 (Ruminococcaceae), OTU-182190 (Lachnospiraceae), OTU-470114 (*Clostridium leptum*), OTU-182190 (*Clostridium*) and OTU-175682/109018 (*Eubacterium rectale*) were the top six OTUs positively associated with mitochondrial proteins expression. Among the OTUs displaying negative correlations, OTU-529659 (*A. parvulum*), OTU-570199 (*Vagococcus*), OTU-158660 (*Bacteroides*), OTU-353985 (*Parabacteroides distasonis*) and OTU-64396 (*Fusobacterium*) were identified as the top candidates exhibiting reverse correlations with up to 31 mitochondrial proteins including proteins from the respiratory chain and the H_2_S detoxification complex.

### *A. parvulum* causes pancolitis in *Il10* ^*−/−*^ mice

**Figure 4:**
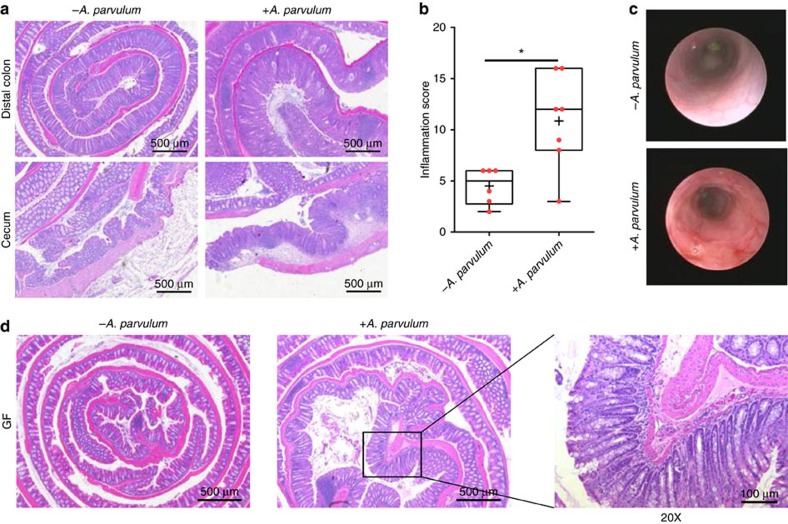
*Atopobium parvulum* induces rapid and severe pan-colitis in *Il10*^*−/−*^ mice. (**a**) Representative histological sections of the cecum and colon of *Il10*^*−/−*^ mice under specific pathogen free (SPF) conditions that were either associated with (+) or without (−) *A. parvulum*. (**b**) Blinded histological score of inflammation (*n*=6 to 7 per group; crosses indicate the mean while horizontal lines indicate the median; comparison by Mann–Whitney two-tailed test; *P<0.05). (**c**) Macroscopic monitoring of inflammation with a murine endoscope (data is representative of two animals). (**d**) Representative histological sections of the distal colon of germ-free (GF) *Il10*^*−/−*^ mice mono-associated with or without *A. parvulum*.

Altogether these observations suggest that *A. parvulum* might play a key role in the development or progression of inflammation severity in the CD patients. To evaluate the colitogenic potential of *A. parvulum*, we utilized colitis-susceptible *Il10*^*−/−*^ mice^18^,^19^. Germ-free *Il10*^*−/−*^;NF-κB^EGFP^ mice were transferred to specific pathogen free (SPF) housing and after 2 weeks acclimatization, gavaged with *A*. *parvulum* (10^8^ colony-forming units (CFUs) per mouse). Thereafter the mice were gavaged with *A. parvulum* weekly for 6 weeks and killed 6 weeks later. Compared with control uninfected *Il10*^*−/−*^;NF-κB^EGFP^ mice, *A*. *parvulum*-colonized *Il10*^*−/−*^ mice displayed colitis (crypt hyperplasia, ulcers, goblet cell depletion and immune cell infiltration) with histologic inflammation scores significantly higher compared with controls (Mann–Whitney *U-*test *P*<0.05; Fig. 4a,b). Colonoscopy imaging revealed mucosal erythema, friability and mucosal ulceration in *A*. *parvulum*-colonized *Il10*^*−/−*^ mice compared with the healthy mucosa observed in control mice (Fig. 4c). At the molecular level, the colon of *A*. *parvulum*-colonized *Il10*^*−/−*^ mice showed increased expression of *Cxcl1* and *Il17* compared with controls (Mann–Whitney *U*-test *P*<0.01; Supplementary Fig. 14). To determine whether the colitogenic effect of *A. parvulum* was dependent on the presence of commensal bacteria, we mono-associated germ-free *Il10*^*−/−*^ mice with *A. parvulum* for 8 weeks. Interestingly, *A. parvulum* mono-associated GF *Il10*^*−/−*^ mice did not cause significant colitis compared with controls suggesting that this bacterium cannot induce disease on its own (Fig. 4d; Histological score: 1.6 versus 0.67 respectively; not significant).

**Figure 5:**
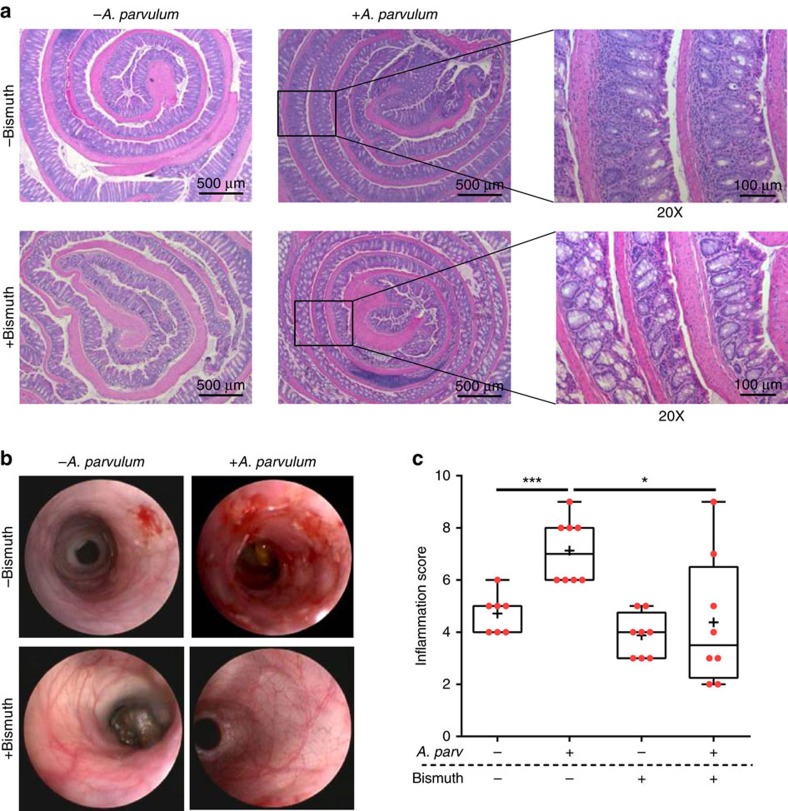
Induction of *A. parvulum-*associated colitis requires the gut microbiota and hydrogen sulfide. (**a**) Representative histological Swiss-rolled sections of the colon of *Il10*^*−/−*^ mice associated (+) or not associated (−) with *A. parvulum*, treated (+) or not treated (−) with bismuth and kept under specific pathogen free (SPF) conditions for 14 weeks. (**b**) Representative murine endoscopies of SPF *Il10*^*−/−*^ mice associated (+) or not-associated (−) with *A. parvulum*, treated (+) or not-treated (−) with bismuth. (**c**) Blinded inflammation scores (*n*=7 to 8 per group) for *Il10*^*−/−*^ mice under SPF conditions; crosses indicate the mean while horizontal lines indicate the median; two-tailed Mann–Whitney test was used for statistical comparison; *P<0.05, ***P<0.001.

Bismuth (III)-subsalicylate (hereafter denoted as ‘bismuth') is a known H_2_S scavenger^20^. To test the potential role of H_2_S in *A. parvulum*-mediated colitis in *Il10*^*−/−*^ mice, gnotobiotic *Il10*^*−*/*−*^ mice were transferred to SPF conditions and randomized into four groups; SPF only, SPF plus *A*. *parvulum*, SPF plus bismuth and finally, SPF plus *A. parvulum* and bismuth. As expected *A. parvulum* worsened the development of colitis (Fig. 5a–c). Interestingly, treatment with bismuth significantly improved the colitis scores in *A. parvulum*-colonized mice (Fig. 5a–c) and prevented expression of *Il1β*, *Il12p40* and *Cxcl1* messenger RNA (mRNA; Supplementary Fig. 15). Although these findings suggest a role for H_2_S production in *A. parvulum*-induced colitis, the reduced colitis observed with bismuth administration could also be due to bismuth's potential antimicrobial activity on *A. parvulum* and/or the intestinal microbiota.

## Discussion

In this study, we found key alterations in the microbiota–host interplay in newly diagnosed children with IBD providing insight into the mechanism of IBD pathogenesis and potentially opening new avenues for treatment interventions. Our study found that mitochondrial proteins were the primary proteins downregulated in CD indicating a central role for the mitochondria in CD pathogenesis. The significant downregulation of these proteins was associated with a depletion of butyrate producers together with a bloom of pathobionts many of which are known to be potent H_2_S producers. These results suggest a regulatory relationship between the mitochondria and microbiota and a disturbance of this relationship in the CD patients.

Accumulating data support a role for mitochondrial dysfunction in IBD pathogenesis. Reduced mitochondrial functions have been reported in UC patients with an up to 60% decreased activity of the respiratory chain complexes II, III and IV (ref. 21). Morphological changes in the mitochondria have been observed in the IBD patients^22^,^23^,^24^ and functional defects at complexes III and IV have been described in a paediatric CD patient^25^. Variants in mitochondrial DNA, which result in increased metabolic activities, protect mice from colitis^26^. The decreased expression of the mitochondrial proteins observed here in paediatric CD patients would perturb mitochondrial functions. Such mitochondrial perturbations have been shown to result in increased epithelial permeability through ROS production and promote transcytosis of bacteria across the epithelial layer^27^. Increased intestinal permeability has been reported in active CD further suggesting a role for mitochondrial dysfunction in the pathogenesis of IBD^28^,^29^.

Butyrate is the main energy source of colonocytes and is produced by the bacterial fermentation of unabsorbed carbohydrate. Here, we demonstrate that the intestinal microbiota of paediatric CD patients is characterized by a significant decrease in the relative abundance of butyrate producers as has been reported previously in the context of paediatric IBD^3^. Our data also revealed a positive correlation between the relative abundance of the butyrate producers and mitochondrial proteins, suggesting a signalling role for butyrate in host mitochondrial gene expression. In support of this observation, butyrate is known to activate the expression of the genes encoding the host mitochondrial H_2_S detoxification components^30^ and our proteomic analyses indicate a diminished capacity for H_2_S detoxification by paediatric CD patients. Therefore, we postulate that the depletion of butyrate-producing microbes from the colonic microbiota may dampen host H_2_S defense systems. This ‘impaired' host would be highly susceptible to further damage caused by enhanced H_2_S production, resulting in additional metabolic stress and subsequently increased mucosal inflammation. Notably, this decrease in butyrate producers is associated with a significant increase in pathobionts, many of which are known to produce H_2_S. These include *Fusobacterium*, *Veillonella* and *Atopobium parvulum* whose relative abundance correlated negatively with the expression of the mitochondrial proteins. Butyrate exhibits anti-inflammatory properties, whereas H_2_S is an important mediator of many physiological and pathological processes and has been previously associated with UC and colorectal cancer^17^,^31^. Interestingly, *A. parvulum* was identified by network analysis as the most prominent microbe associated with mitochondrial dysfunction. Importantly, the increased abundance of *A. parvulum* as a function of inflammation severity in paediatric CD patients was not observed in UC and therefore is not simply a consequence of inflammation, suggesting a causal effect in CD.

Although causal relationships would be better identified by monitoring changes in microbiota composition before the onset of the disease while following disease establishment, logistical constraints preclude this experiment. Instead we tested the hypothesis that changes in microbial composition between distinct and progressive stages of the disease would unravel causal interactions among the host and specific members of the gut microbes. Indeed, the observation that the relative abundance of *A. parvulum* correlates positively with inflammation severity and negatively with both the mitochondrial protein expression and the level of butyrate producers prompted us to assess the colitogenic potential of *A. parvulum*. Interestingly, we found that *A. parvulum* triggers the development of colitis in *Il10*^*−/−*^ mice under specific pathogen-free conditions but not under mono-association conditions. These results indicate a key role for both *A. parvulum* and the gut microbiota in colitis development. In support to our findings, a recent report on CD patient response to exclusive enteral nutrition (EEN) therapy has reported that a putative *A. parvulum* OTU was enriched in CD patients before treatment and was negatively associated with days on EEN^32^. Although a role for H_2_S production by *A. parvulum* in colitis development remains to be proven directly, excess H_2_S has been recently shown to act as an autocrine T-cell activator^33^, potentially contributing to unwanted T-cell responses against commensal bacteria, consistent with our observation that the intestinal microbiota is required for *A. parvulum*-induced experimental colitis. Accordingly, potential H_2_S trapping by bismuth may have contributed to the mitigation of *Atopobium*-induced colitis development in our mouse model. This finding is in agreement with a prospective mechanistic role for H_2_S in inflammation. A role for H_2_S in colitis development is supported by the recent finding that microbial H_2_S formation contributes to mucus degradation opening the intestinal barrier to toxic compounds and pathobionts^34^.

H_2_S is an important mediator of many biological processes (angiogenesis, cytoprotection, metabolism, inflammation) and can be pro- or anti-inflammatory depending on its concentration and the particular circumstance^35^,^36^. In light of these opposite effects, a recent study reported that paediatric CD patients undergoing EEN treatment and presenting with reduced colonic inflammation had increased levels of fecal H_2_S^37^. The findings from the latter study challenge the concept of a pro-inflammatory role for H_2_S in CD. However, as EEN feed is enriched in sulfur-containing amino-acids, the observed increase in H_2_S levels might simply be a consequence of a change in diet. Clearly, complex modes of action of H_2_S need to be further explored to reconcile these apparent controversial observations. Furthermore, the impact of bacterial-derived exogenous H_2_S on the intestine may be quite different than endogenous cell-based H_2_S.

Altogether, our results emphasize the importance of the microbial community and its interaction with the host in the pathogenesis of IBD. We propose a central role for mitochondrial dysfunction in colitis development. Although it is difficult to identify the cause underpinning observed downregulation of mitochondrial proteins, the loss of butyrate producers in paediatric CD patients will lead to decreased butyrate production which in turn will impair mitochondrial functions likely resulting in ROS production, increased epithelial permeability and translocation of commensal microbes across the epithelial barrier. Intriguingly, antibiotic exposure has been shown to reduce the abundance of butyrate producers^3^ and also markedly increases the risk of CD in children (odds ratio 2.75, 95% confidence interval 1.72–4.38; ref. 38). We have shown that *A. parvulum* causes colitis in a susceptible mouse model and that the host microbiota is required for colitis development. Therefore, the increased abundance of *A. parvulum* in paediatric CD patients with severe inflammation suggests a key role for this microbe in driving mucosal inflammation and disease exacerbation. Our results point toward a mechanism of CD pathogenesis involving a disruption of the mitochondria–microbiota relationship leading to dysfunctional mitochondria in the presence of microbes themselves involved in a complex interactive microenvironment at the MLI required for mucosal inflammation.

## Methods

### Ethics statement

The collection of samples from paediatric patients was approved by the Research Ethics Board of the Children's Hospital of Eastern Ontario (CHEO). The protocol for the use of *Il10*^*−/−*^ mice was approved by the Institutional Animal Care and Use Committee of University of North Carolina at Chapel Hill and the University of Florida. Written informed consent was obtained from the participants or parents.

### Study design and sampling

We conducted a cross-sectional study of all eligible patients under 18 years of age scheduled to undergo colonoscopy for their initial diagnostic work-up for suspected IBD. Those who were not diagnosed with IBD acted as controls. Exclusion criteria relating to known conditions affecting the intestinal microbiota composition included: (1) a body mass index greater than the 95th percentile for age; (2) diabetes mellitus (insulin and non-insulin dependent); (3) infectious gastroenteritis within the preceding 2 months; and (4) use of any antibiotics or probiotics within 4 weeks before colonoscopy, or (5) irritable bowel syndrome. These same exclusion criteria were applied to the non-IBD control group. All IBD cases met the standard diagnostic criteria following thorough clinical, endoscopic, histologic and radiological evaluation^39^. Disease location was based on endoscopic and radiologic appearance and characterized using the Paris modification of the Montreal Classification for IBD^40^. Clinical disease activity of CD was determined using the PCDAI^41^ and of UC using the PUCAI (Pediatric Ulcerative Colitis Activity Index)^42^. All the controls had macroscopically and histologically normal mucosa, and did not carry a diagnosis for any known chronic intestinal disorder (for example, celiac disease, eosinophilic enterocolitis, irritable bowel syndrome). Subject clinical data were collected and managed using REDCap (Research Electronic Data Capture) hosted at the CHEO Research Institute. REDCap is a secure, web-based application designed to support data capture for research studies^43^.

Mucosal–luminal interface samples were collected from the right (ascending) colon (RC); *n*=94, 63 and 37 for CD, control and UC, respectively at the time of diagnostic colonoscopy. Colonoscopy preparation was done as per standard protocol modified to one day^44^. During colonoscopy, once the cecum and proximal ascending colon was reached, any loose fluid and debris was aspirated. Thereafter, sterile water was flushed onto the mucosa to dislodge the mucus layer from mucosal epithelial cells and the mixture of water, mucus and intestinal cells was aspirated into a sterile container through the colonoscope. These samples were immediately placed on ice in the endoscopy suite and immediately transferred to the lab for processing and storage at −80 °C. In addition, mucosal biopsies (*n*=21 and 10 for CD and control, respectively) were collected from macroscopically involved and non-involved (if the latter were identified) areas of the mid right colon. The biopsies were flash-frozen on dry ice in the endoscopy suite and immediately stored at −80 °C until further processing.

### Metagenomic DNA extraction

Metagenomic DNA was extracted using the Fast DNA Spin Kit (MP Biomedicals, Solon, OH, USA) and a FastPrep-24 instrument (MP Biomedicals) with two mechanical lysis cycles at speed 6.0 m s^−1^ for 40 s, with 5 min cooling on ice between the two cycles. Next, the DNA was isolated and purified as per the Fast DNA Spin Kit protocol. The extracted DNA was quantified by Qubit fluorometer (Invitrogen, Carlsbad, CA, USA) and stored at −20 °C until use.

### 16S rDNA-V6 library construction for Illumina sequencing

The V6 region of 16S rDNA was amplified using two successive PCR reactions. The first PCR added the Illumina paired-end sequencing adaptors and the barcode sequences using modified universal 16S rDNA-V6 primers^45^ (Supplementary Table 2, Supplementary Fig. 16). Each reaction was prepared in a total volume of 50 μl using 50 ng of the extracted DNA, 0.5 μM of each primer and 1 × Phusion Flash High-Fidelity PCR Master Mix (Thermo Scientific, Vilnius, Lithuania). The reaction was heated to 98 °C for 30 s and then subjected to 10 cycles of 98 °C for 5 s, 61 °C for 15 s with 1 °C drop each cycle and 72 °C for 15 s, followed by additional 15 cycles with an annealing temperature of 51 °C for 15 s and a final extension at 72 °C for 5 min. The second PCR was carried out using 10 μl of the first PCR products in a final volume of 50 μl using the primers PCRFWD1/PCRRVS1 (Supplementary Table 2). The PCRFWD1/PCRRVS1 primers are complementary to the flow cell primers at the 5′ end and Illumina paired-end sequencing adaptor at their 3′ end. The second PCR conditions were 1 min at 98 °C, 15 cycles of 10 s at 98 °C, 30 s at 65 °C and 30 s at 72 °C followed by a final extension step at 72 °C for 5 min. The amplicons of each sample were visualized on 1.5% agarose gel and purified using a Montage PCR_96_ Cleanup Kit (Millipore, Billerica, MA, USA). Afterwards, the DNA concentration in each reaction was quantified using the Qubit fluorometer (Invitrogen, Carlsbad, CA, USA) according to the manufacturer's instructions. Finally, equimolar quantities of the amplicon from all the samples were pooled, gel purified using a QIAquick Gel Purification Kit (Qiagen, Hilden, Germany) and sent to The Center for Applied Genomics, Toronto, for 100 bp paired-end Illumina HiSeq2500 sequencing.

### Illumina sequencing of pure culture bacterial cultures

Pure cultures of various bacterial species (*Actinomyces odontolyticus* ATCC 17424, *Campylobacter jejuni* NCTC 11168, *Enterococcus faecalis* ATCC 14433, *Fusobacterium nucleatum* ATCC 25586, *Lactobacillus rhamnosus* ATCC 7464, *Streptococcus cristatus* clinical isolate) were grown as required under either anaerobic, microaerophilic (10% O_2_, 5% CO_2_, 85% N_2_) or aerobic conditions at 37 °C. DNA for each sample was extracted using the Fast DNA Spin Kit as described above (hereafter referred to as ‘pure' samples) and Sanger sequenced to confirm the identity of each bacterial species. In addition, aliquots of the extracted DNA were repurified a second time using the FastDNA kits to assess the impact of potential kit contamination (hereafter referred to as ‘re-extract' samples). 16S rDNA-V6 libraries for all the samples were constructed, with triplicate libraries constructed for the re-extract samples and sent for Illumina sequencing as described above.

### Illumina microbiota sequencing data analysis

Raw data were first analysed for decreasing quality along the read. As expected, we noted decreasing quality near the end of the reads. Given that our paired end reads are expected to overlap by ∼21–25 nt (Supplementary Fig. 16), we trimmed four nucleotides from the 3′ end of the sequences using the fastx_trimmer script (http://hannonlab.cshl.edu/) to remove error-prone bases that could result in incorrect read merging. To note, this trimming step reduced the total number of singletons/doubletons in the final rarefied OTU table by 5% (data not shown). The trimmed paired-end sequences were merged using Fast Length Adjustment of Short reads^46^. More than 95% of the reads were successfully merged, while the sequences that failed to merge were discarded. The merged reads were then quality filtered with a minimum quality score of 20 over 90% of the sequence using the fastq_quality_filter command from the Fastx toolkit (http://hannonlab.cshl.edu/). High-quality reads were demultiplexed according to the forward and the reverse barcode sequences and the barcode trimmed using NovoBarCode (Novocraft.com). Afterwards, the reads were converted from fastq to fasta and were fed to QIIME 1.8.0 (ref. 47) to determine the taxonomic and diversity profiles of the samples. First, reads were clustered into OTUs using a closed-reference OTU picking workflow against the Greengenes reference set (release 4 February 2011) based on an average percentage of identity of 97%. Note that the same Greengenes database was used for the Illumina, Ion Torrent and 454 pyrosequencing data sets discussed below. Singletons and doubletons were removed and a table of OTU counts per sample was generated. Next, the OTU table was randomly subsampled to a total number of reads per sample of 200,000. The resulting rarefied OTU table was used to analyse the microbiota structure and diversity using the microbial ecology tools available in the QIIME package and as input for all other downstream analyses. Any further analysis-specific filtering of the input OTU table is described within each individual ‘Methods' section. The phylogenetic tree was constructed using the FastTree method of QIIME 1.8.0 and the generated file was exported to the Interactive Tree of Life (iTOL) software^48^,^49^ to view and format the constructed tree.

### Illumina pure culture sequencing data analysis

The raw reads obtained for the pure culture sequencing experiments were processed as described above for the Illumina metagenome analysis to generate an OTU table rarefied to 200,000 reads per sample and analysed in phyloseq^50^. Each pure culture consisted of single ‘dominate' OTU representing with a median of >99% of the total reads and with a taxonomic classification matching the expected bacteria at the genus level (Supplementary Fig. 5, Supplementary Data 5). Any OTUs present with a taxonomic classification matching the expected bacteria at the family level were considered to be potential sequencing errors. The reads that were matched against these erroneous OTUs were extracted and re-aligned using BLASTN against the dominant OTU to determine the PCR/sequencing error rate (Supplementary Fig. 6, Supplementary Data 5). OTUs present in >90% of the samples (in either the pure or re-extract sample sets) were considered to be background contamination originating from either the DNA extraction kits or reagents used during PCR amplification (Supplementary Fig. 5).

### Library construction for Ion Torrent sequencing

The V4 or V6 region of 16S rRNA was amplified in a single PCR reaction that incorporated both the Ion Torrent adaptors and an 11 bp barcode sequence using modified universal 16S rDNA-V4 (ref. 51) and V6 (ref. 45) primers (Supplementary Table 3). Note that the conserved V6 sequence used for the Ion Torrent sequencing is identical to those used in the 16S rDNA-V6 Illumina library construction described above. Each reaction was prepared in a total volume of 50 μl using 50 ng extracted metagenomic DNA, 0.5 μM of each primer, and 1 × Phusion Flash High-Fidelity PCR Master Mix. For the V6 reactions, the PCR cycling conditions were identical to the first PCR reaction described in the 16S rDNA-V6 Illumina library construction ‘Methods' section. For the V4 reactions, the reaction was heated to 98 °C for 30 s and then subjected to 34 cycles of 98 °C for 30 s, 55 °C for 30 s and 72 °C for 90 s, followed by a final extension at 72 °C for 5 min as previously described^51^. The samples were purified using Purelink PCR purification columns and quantified using a Qubit fluorometer (Life Technologies). PCR products were visualized on a 1.5% agarose gel to ensure successful amplification and that the products were the expected size. Two hundred nanograms of each sample was subsequently pooled together for both the V4 and V6 libraries. The pooled libraries were size-selected using Agencourt AMPure XP DNA purification beads to remove primer dimers. The molar concentrations of each library pool were determined using an Agilent Bioanalyzer and the V4/V6 libraries were combined in an equimolar ratio. This equimolar V4/V6 pool was subjected to emulsion PCR using the Ion OneTouch HiQ Template Kit and sequencing of the templated beads was completed on a single Proton Chip on an Ion Torrent Proton using an Ion Proton HiQ sequencing kit according to the manufacturer's instructions (Life Technologies).

### Ion Torrent sequencing data analysis

Ion Torrent sequencing was done for both the V4/V6 regions from 36 right colon samples (*n*=13, 12, 11 for control, CD and UC). The unaligned BAM file generated by the Ion Torrent Proton (note that adaptor sequences/primer dimers are automatically removed by the Proton analysis pipeline) was downloaded, converted to fastq format and reads split according to the expected sizes for the V4/V6 regions plus the barcodes (234–266 and 162–194, respectively, representing a ±16 bp window), using samtools^52^ in conjunction with bioawk (https://github.com/lh3/bioawk). The reads were quality trimmed using the modified Mott trimming algorithm implemented in seqtk (https://github.com/lh3/seqtk) and reads that were shorter than the expected size for either the V4 or V6 regions (as described above) after trimming were discarded. The V4/V6 reads were then demultiplexed using FastqMultX^53^, converted from fastq to fasta and fed to QIIME 1.8.0^47^ to determine the taxonomic profiles of the samples. The reads were clustered into OTUs using a closed-reference OTU picking workflow against the Greengenes reference set (release 4 February 2011) based on an average percentage of identity of 97%. The singletons/doubletons were removed from both the V4/V6 OTU tables and the number of reads/sample was normalized across sample groups (100,000 reads per sample for V4 and 200,000 reads per sample for V6). The rarefied tables were used to calculate alpha/beta diversities in phyloseq^50^ and to identify the microbial biomarkers for each tested category using the linear discriminant analysis effect size algorithm^11^ with the default parameters. To compare the Ion Torrent sequencing results to the Illumina results, the Illumina V6 data from the samples subjected to Ion Torrent sequencing was extracted from the Illumina rarefied OTU table (note that the Illumina V6 and Ion Torrent V6 samples were rarefied to the same depth) and analysed as described above for the Ion Torrent data.

### 16S rDNA-V6 454-pyrosequencing

The 454-pyrosequencing sequencing library of the 16S rRNA-V6 region was constructed using a two-step PCR strategy using the primers; 16SF 5′-AAACTCAAAKGAATTGACGG-3′ and 16SR 5′-ACGAGCTGACGACARCCATG-3′ (ref. 45). Ten replicates from each sample were amplified in a 25 μl reaction containing 50 ng DNA template, 1 × PCR buffer, 2 mM MgCl_2_, 0.2 mM dNTPs mix, 0.2 μM of each primer and 2.5 U Platinum Taq polymerase (Invitrogen, Carlsbad, CA, USA). The reactions were heated to 95 °C for 5 min, followed by 15 cycles of 94 °C for 40 s, 46 °C for 1 min and 72 °C for 30 s, and a final extension at 72 °C for 5 min. The amplicons from each sample were pooled and purified with Qiagen's MiniElute PCR purification columns (Qiagen, Hilden, Germany) following its standard protocol and eluted in 25 μl molecular biology-grade water. Two microlitres of the pooled purified amplicons were then used as a template for the second PCR following the same PCR conditions except using 30 amplification cycles instead of 15 cycles. The second PCR added the titanium tails to the 16S-V6 amplicons, which are essential for the 454 sequencing procedure. The amplicons from the second PCR were pooled and purified again using Qiagen MiniElute PCR purification columns. Negative control reactions (no DNA template) were included in all the experiments. PCR products were visualized on a 1.5% agarose gel to check the amplification success. The purified amplicons from different samples were normalized to the same concentration (100 ng μl^−1^), captured to streptavidin coated sepharose beads and exposed to emulsion PCR. Afterwards, the immobilized double stranded amplicons were denatured into single-stranded DNA, which was then annealed to the sequencing primer by heating at 65 °C for 5 min. The single-stranded DNA-containing beads were sequenced on a 454 Genome Sequencer FLX System (Roche Diagnostics GmbH) using GS Titanium chemistry according to the standard amplicon sequencing protocol. Samples covering the three tested phenotypes (two to four samples each) were sequenced on the same run with each being sequenced in a 1/16 section of 70 × 75 picotiter plate. Note that as the samples were physically separated from each other during the sequencing reaction, there was no need to use barcoded primers.

### 454-pyrosequencing data analysis

A total of 346,160 reads were generated from the 454 pyrosequencing of 16S rDNA-V6 region from 26 right colon samples. The raw pyrosequencing reads were de-noised and processed to remove low quality and short reads using Quantitative Insights Into Microbial Ecology pipeline release 1.8.0 (QIIME 1.8.0; ref. 47) according to the following parameters: (1) minimum read length of 100 bp, (2) exact matching to the sequencing primers, (3) no ambiguous nucleotides and (4) a minimum average quality score of 20. Next, sequences were *de novo* clustered using UCLUST^54^ based on average percentage of identity of 97%. The most abundant read from each OTU was picked as a representative sequence for that cluster. The representative sequences were then aligned using PyNAST with a minimum alignment length of 100 and a minimum percentage identity of 75%, followed by checking the chimeric OTUs with the blast_fragments approach. Only six representative sequences were identified as chimeric and therefore were removed. Taxonomy assignments were made with BLAST^55^ by searching the representative sequences against the Greengenes database (release 4 February 2011) with an e value <1e−8 and a confidence score of ≥0.5. Next, singletons (OTUs that had only one matching sequence) were filtered out from the resulting OTU table. The OTU table was then used to determine the alpha and beta diversity within and between the samples using the QIIME'S default criteria. To identify the microbial biomarkers for each tested category, the relative abundance of different phylogenetic levels computed by QIIME was analysed by linear discriminant analysis effect size algorithm^11^ using its default parameters. The 454-pyrosequencing reads assigned as *Atopobium* by QIIME analysis were retrieved and found to match to *A. parvulum* following alignment of the reads against the RDP^56^ and NCBI databases (the aligned region covered the entire 454 sequence length with >99% sequence identity to *A. parvulum*).

### Statistical analysis of the microbiota data

Several statistical approaches were used to identify taxa significantly associated with disease status and severity. Identification of OTUs exhibiting differential abundance between patients with different disease severity (mild versus moderate versus severe) was performed using the metagenomeSeq R package—a statistical approach that controls for confounding factors and accounts for undersampling^12^. Briefly, the OTU counts were normalized using a cumulative sum scaling approach and OTUs with a normalized count <5 in at least one of the groups were discarded. OTUs exhibiting differential abundance as a function of disease severity were identified using a zero-inflated Gaussian mixture model by incorporating gender and the inflammation status of the ascending colon (inflamed or not inflamed) as confounding covariates. Differentially abundant OTUs were selected according to the following algorithm: (1) OTU present in more than 50% of the samples from at least one group; (2) a fold change in relative abundance ≥2 and (3) a statistical significance with an adjusted *P* value (calculated using the Benjamini–Hochberg method) <0.05. A Kruskal–Wallis test with a *post hoc* Dunn's test was performed to compare the relative abundance of specific taxa or groups of bacteria as a function of disease status (CD versus UC versus control) and disease severity (mild versus moderate versus severe). The relative abundance of the taxa identified was also analysed by principal coordinate analysis (PCoA). All the statistical analyses were performed using XLSTAT (Addinsoft, NY, USA) and/or GraphPad Prism version 6 (GraphPad, La Jolla, CA, USA).

### Validation of V6-16S sequencing

The quantification of *A. parvulum* and sulfate reducing bacteria relative to total bacteria was determined by conducting quantitative PCR (qPCR) on the extracted metagenomic DNA using an Applied Biosystems 7,300 and primers listed in Supplementary Table 4. Each sample was tested in duplicate in a total volume of 25 μl per reaction. Fifty nanograms of template DNA was added to a reaction mixture containing 0.3 μM of each primer (0.5 μM for 16S rDNA universal primers) and 1 × QuantiFast SYBR Green PCR master mix (Qiagen, Hilden, Germany). The amplification conditions were 5 min at 95 °C followed by 40 cycles of 95 °C for 10 s and 66 °C for 1 min with data collection at the second step of each cycle. Ct values were then extracted using the Applied Biosystems 7300 software version 1.3.1 and the relative abundance of each taxon was calculated as a *Δ*Ct value (taxon Ct–universal 16S rRNA Ct). To validate the specificity of Apar-711F and Aparv-881R, fresh PCR amplicons from total DNA extracted from two different mucosal aspirates was cloned using the TOPO TA cloning vector (Invitrogen, Carlsbad, CA, USA) according to the manufacturer's instructions. Next, the plasmid containing the 16S rDNA gene fragment was extracted from six different clones by the QIAprep Spin Miniprep kit (Qiagen, Hilden, Germany) followed by capillary sequencing using M13F and M13R primers.

### Quantification of butyrate-producers by qPCR

The overall abundance of butyrate-producing bacteria was determined by quantifying the amount of the butyryl CoA:acetate CoA-transferase (BCoAT) gene using the primers BCoATscrF/BCoATscrR (Supplementary Table 4) as described elsewhere^57^,^58^. The BCoAT gene was amplified from 50 ng of metagenomic DNA in a 25 μl qPCR reaction containing 1 × QuantiTect SYBR Green PCR master mix (Qiagen) and 2.5 μM of BCoATscrF/BCoATscrR primers. The amplification conditions were as follows: one cycle of 95 °C for 15 min; 40 cycles of 94 °C for 15 s, 53 °C and 72 °C each for 30 s with data acquisition at 72 °C. The 16S rDNA gene was used to normalize total bacterial content in each sample and was amplified using adapted universal primers UniF/UniR (Supplementary Table 4)^59^. The assay was done in duplicate for each sample. Delta-Ct (*Δ*Ct) for each target was calculated by subtracting the Ct of the 16S rDNA from the BCoAT Ct. Then, the *Δ*Ct values were compared between the CD and control groups using a Mann–Whitney two-tailed test with a Dunn's *post hoc* test and *P* values below 0.05 were considered significant.

### Microbial correlation network analysis

A correlation network was constructed to identify co-occurrence and mutual exclusion among OTUs from the control subjects and CD patients. To focus on bacteria potentially associated with disease, only the OTUs exhibiting differential abundance as a function of disease severity were chosen for this network analysis. In addition, OTUs that were found to be absent in more than two-third of the samples or with a minimum occurrence <15 across all the samples were removed from the data set, leaving a matrix of 97 OTUs. The network was built as previously described^60^ using the CoNet Cytoscape plug-in. The correlation scores were calculated for each OTU pair using a combination of two correlation (Pearson and Spearman) and two dissimilarity measures (Bray–Curtis and Kullback–Leibler). The 400 top- and bottom-ranking edges from each method were retrieved. Edge- and method-specific permutations (100) were used to control for potential false-positive correlations. The resulting distribution was used to calculate statistical significance for each edge using 100 bootstrap iterations. The *P* value was adjusted for multiple comparisons using a Benjamini–Hochberg correction and a value <0.05 was deemed significant. The *P* value was obtained for each correlation or dissimilarity measure and only edges supported by at least two significant measures were included. The final network consisted of 89 nodes (OTUs) and 341 edges (correlations). The resulting network was visualized using Cytoscape 3.2.1 (ref. 61).

### Stable isotope labelling by amino acids in cell culture

The human hepatic HuH7 cells (HuH-7) and human embryonic kidney 293 cells (HEK-293) were originally acquired from ATCC and the human colorectal cancer 116 cells (HCT-116) were obtained from the Japanese Collection of Research Bioresources Cell Bank. HuH-7, HEK-293 and HCT-116 were individually grown at 37 °C in a 5% CO_2_ humidified incubator (to note, all three cell lines used in this study were found to be mycoplasma positive). Stable isotope labelling by amino acids in cell culture (SILAC) medium was prepared as follows: DMEM lacking lysine, arginine and methionine was custom prepared by AthenaES (Baltimore, MD, USA) and supplemented with 30 mg l^−1^ methionine (Sigma-Aldrich; Oakville, ON, Canada), 10% (v/v) dialysed FBS (GIBCO-Invitrogen; Burlington, ON,Canada), 1 mM sodium pyruvate (Gibco-Invitrogen), 28 g ml^−1^ gentamicin (Gibco-Invitrogen), and [^13^C_6_,^15^N_2_]-L-lysine, [^13^C_6_,^15^N_4_]-L-arginine (heavy isotopic form of amino acids; Heavy Media) from Sigma-Aldrich (Oakville, ON, Canada) at final concentrations of 42 and 146 mg l^−1^ for arginine and lysine, respectively. For HCT-116, the concentration of arginine was increased to 84 mg l^−1^. The cells were grown for at least 10 doublings in SILAC media to allow for complete incorporation of the isotopically labelled amino acids into the cells.

### Determination of the rate of SILAC amino acids incorporation

The cells were grown to 80% confluency in SILAC medium (5 × 10^6^ cells were plated in 10-cm dish). Next, the cells were washed twice with ice-cold phosphate-buffered saline and lysed by addition of 1 ml of 1X RIPA buffer (50 mM Tris (pH 7.6), 150 mM NaCl, 1% (v/v) NP-40, 0.5% (w/v) deoxycholate, 0.1% (w/v) SDS with protease inhibitor cocktail (Complete Mini Roche; Mississauga, ON,Canada) and phosphatase inhibitor (PhosStop Roche tablet)). The lysates were then transferred to 15 ml conical tubes and the proteins were precipitated by addition of 5 ml ice-cold acetone followed by incubation at −20 °C overnight. The proteins were collected by centrifugation (3,000*g*, 10 min, 4 °C), washed with ice-cold acetone two times and the protein pellets were re-solubilized in 300 μl of a 50 mM NH_4_HCO_3_ solution containing 8 M urea. The protein concentrations were determined by the Bradford dye-binding method using Bio-Rad's Protein Assay Kit (Mississauga, ON, Canada). For the general in-solution digestion, 200 μg of protein lysates were reconstituted in 50 mM NH_4_HCO_3_ (200 μl) and the proteins were reduced by mixing with 5 μl of 400 mM DTT at 56 °C for 15 min. The proteins were then subjected to alkylation by mixing with 20 μl of 400 mM iodoacetamide in darkness (15 min at room temperature) followed by addition of 800 μl of 50 mM NH_4_HCO_3_ to reduce the urea concentration to ∼0.8 M. Next, the proteins were digested with TPCK-trypsin solution (final ratio of 1:20 (w/w, trypsin:protein) at 37 °C for 18 h. Finally, the digested peptides were desalted using C18 Sep-Pack cartridges (Waters), dried down in a speed-vac, and reconstituted in 0.5% formic acid before mass spectrometric analysis (as described below) and the determination of labelling efficiency. The incorporation efficiency was calculated according to the following equation: (1-1/Ratio(H/L)); where H and L represents the intensity of heavy and light peptides detected by mass-spectrometry, respectively. Labelling was considered complete when values reached at least 95% for each cell type.

### Proteomic analysis of biopsies

Biopsies were lysed in 4% SDS, 50 mM Tris-HCl (pH 8.0) supplemented with proteinase inhibitor cocktail (Roche) and homogenized with a Pellet pestle. The lysates were sonicated three times with 10 s pulses each with at least 30 s on ice between each pulse. The protein concentrations were determined using the Bio-Rad DC Protein Assay. The proteins were processed using the Filter Aided Sample Preparation method as previously described with some modifications^62^. Biopsy lysates (45 μg of proteins) and heavy SILAC-labelled cell lysates (15 μg from each HuH-7, HEK- 293 and HCT-116 cells) were mixed at a 1:1 weight ratio and transferred into the filter. The samples were centrifuged (16,000*g*, 10 min), followed by two washes of 200 μl 8 M urea, 50 mM Tris-HCl pH 8.0. The samples were then reduced by incubation in 200 μl of 8 M urea, 50 mM Tris-HCl (pH 8.0) supplemented with 20 mM dithiothreitol. After centrifugation, the samples were subjected to alkylation by adding 200 μl of 8 M urea, 50 mM Tris-HCl pH 8.0, containing 20 mM iodoacetamide (30 min at room temperature protected from light). The samples were washed twice using 200 μl 8 M urea, 50 mM Tris-HCl pH 8.0 to remove excess SDS. To further dilute urea, two washes of 200 μl 50 mM Tris-HCl pH 8.0 were performed. For the trypsin digest, the samples were incubated in 200 μl of 50 mM Tris-HCl pH 8.0, containing 5 μg of Trypsin (TPCK Treated, Worthington) on a shaker (250 rpm) at 37 °C overnight. Finally, 200 μl of 50 mM Tris-HCl pH 8.0 was added to elute the peptides by centrifugation (twice). The peptides were fractionated, using an in-house constructed SCX column with five pH fractions (pH 4.0, 6.0, 8.0, 10.0 and 12.0). The buffer composition was 20 mM boric acid, 20 mM phosphoric acid and 20 mM acetic acid, with the pH adjusted by using 1 M NaOH. Finally, the fractionated samples were desalted using in-house C18 desalting cartridges and dried in a speed-vac before LC-MS analysis.

### Mass-spectrometry analyses

All resulting peptide mixtures were analysed by high-performance liquid chromatography/electrospray ionization tandem mass spectrometry (HPLC-ESI-MS/MS). The HPLC-ESI-MS/MS consisted of an automated ekspert nanoLC 400 system (Eksigent, Dublin, CA, USA) coupled with an LTQ Velos Pro Orbitrap Elite mass spectrometer (ThermoFisher Scientific, San Jose, CA, USA) equipped with a nanoelectrospray interface operated in positive ion mode. Briefly, each peptide mixture was reconstituted in 20 μl of 0.5% (v/v) formic acid and 12 μl was loaded on a 200 μm × 50 mm fritted fused silica pre-column packed in-house with reverse phase Magic C18AQ resins (5 μm; 200 Å pore size; Dr Maisch GmbH, Ammerbuch, Germany). The separation of peptides was performed on an analytical column (75 μm × 10 cm) packed with reverse phase beads (3 μm; 120 Å pore size; Dr Maisch GmbH, Ammerbuch, Germany) using a 120 min gradient of 5–30% acetonitrile (v/v) containing 0.1% formic acid (v/v) (JT Baker, Phillipsburg NJ, USA) at an eluent flow rate of 300 nl min^−1^. The spray voltage was set to 2.2 kV and the temperature of heated capillary was 300 °C. The instrument method consisted of one full MS scan from 400 to 2,000 *m*/*z* followed by data-dependent MS/MS scan of the 20 most intense ions, a dynamic exclusion repeat count of 2 and a repeat duration of 90 s. The full mass was scanned in an Orbitrap analyzer with R=60,000 (defined at *m*/*z* 400), and the subsequent MS/MS analyses were performed in LTQ analyzer. To improve the mass accuracy, all the measurements in the Orbitrap mass analyzer were performed with on-the-fly internal recalibration (‘Lock Mass'). The charge state rejection function was enabled with charge states ‘unassigned' and ‘single' states rejected. All the data were recorded with Xcalibur software (ThermoFisher Scientific, San Jose, CA, USA).

### Mass-spectrometry database search and bioinformatic analysis

All the raw files were processed and analysed by MaxQuant, Version 1.2.2.5 against the decoy Uniprot-human database (86,749 entries), including commonly observed contaminants. The following parameters were used: cysteine carbamidomethylation was selected as a fixed modification, with methionine oxidation, protein amino (N)-terminal acetylation and heavy proline set as variable modifications. Enzyme specificity was set to trypsin. Up to two missing cleavages of trypsin were allowed. SILAC double labelling (light: K0R0; heavy: K8R10) was set as the search parameter to assess the conversion efficiency. The precursor ion mass tolerances were 7 p.p.m. and the fragment ion mass tolerance was 0.5 Da for MS/MS spectra. The false discovery rate for peptides and proteins was set at 1% and a minimum length of six amino acids was used for peptide identification. The peptides file was imported into Perseus (version 1.2.0.17) to extract the lysine- and arginine-containing peptides, respectively. The protein-group file was imported into Perseus (version 1.3.0.4) for statistical analysis. Differences between control and CD protein expression were determined using a *t*-test with an adjustment for multiple comparisons (Benjamini–Hochberg method; false discovery rate =0.05) and *q* values of less than 0.05 were considered significant. Kyoto Encyclopedia of Genes and Genomes (KEGG) pathway analysis was achieved using the DAVID Bioinformatics Resources (http://david.abcc.ncifcrf.gov/). DAVID statistical analyses were performed against the whole genome. Proteomics has a tendency to oversample proteins from the cytosol and nucleus while under-sampling membrane-associated proteins. Therefore, the results from DAVID were re-tested against the set of proteins that were not changing in our data set to eliminate any pathway/GO enrichment biases.

### Total RNA extraction from intestinal mucosal aspirates

RNA integrity was preserved by adding an equal volume of RNAlater (Ambion) to the mucosal aspirates before freezing at −80 °C. The frozen aliquot (2 ml) was thawed on ice and the total RNA was extracted following the hot phenol protocol described previously^63^. Briefly, 4 ml of each sample in RNAlater were pelleted by centrifugation at 13,000 × *g* for 5 min at 4 °C. The pellets were washed twice by resuspension in 50% RNAlater/PBS buffer and centrifugation at 13,000*g* for 5 min at 4 °C. Next, the pellets were resuspended and lysed in 2 ml denaturing buffer (4 M guanidium thiocyanate, 25 mM sodium citrate, 0.5% *N*-laurylsarcosine and 1% *N*-acetyl cysteine, 0.1 M 2-mercaptoethanol). The lysate was divided into 500 μl aliquots, to which 4 μl of 1 M sodium acetate (pH 5.2) was added. Each aliquot was then incubated with 500 μl of buffer saturated phenol (pH 4.3) at 64 °C for 10 min with intermittent mixing. The tube was placed on ice for 10 min followed by centrifugation at maximum speed for 30 min at 4 °C. One millilitre of chloroform was added to the aqueous layer and incubated for 15 min on ice, followed by centrifugation at 18,000*g* for 30 min at 4 °C. Afterwards, RNA was precipitated from the aqueous layer by the addition of one-tenth volume 3 M sodium acetate, 500 mM DEPC-treated EDTA and two volumes of cold ethanol followed by overnight incubation at −80 °C. Later, the RNA was pelleted by centrifugation at maximum speed for 30 min at 4 °C, aspirated with 80% cold ethanol and resuspended in 100 μl of RNAse free dH_2_O. The extracted RNA was treated twice with Dnase I (Epicentre) followed by PCR amplification using the 16S rDNA universal primers; Bact-8F and 1391-R (ref. 64), to confirm the absence of genomic DNA. The quality and the quantity of the extracted RNA were determined by NanoDrop 2000 spectrophotometer (ThermoScientific) and confirmed by Bio-Rad's Experion StdSens RNA system according to the manufacturer's instructions and stored at −80 °C until use.

### Quantification of H_2_S detoxification genes expression level

The quantification of the expression level of *TST*, *SQRDL* (Sulfide Quinone Reductase Like) and *COX4-1* (Cytochrome C oxidase subunit IV isoform 1) relative to *hGAPDH* (Glyceraldehyde-3-Phosphate Dehydrogenase) was determined using an Applied Biosystems 7300 and Quantitect SYBR Green RT-PCR kit (Qiagen, Hilden, Germany). The primers used were either designed using the NCBI Primer-BLAST tool^65^ or extracted from the literature (Supplementary Table 5). The specificity of the primers was confirmed by TOPO TA cloning and capillary DNA sequencing as previously mentioned in the qPCR validation of V6-16S sequencing methods section. Each reaction contained 100 ng RNA template, 0.5 μM of each primer, 1 × Quantitect SYBR Green RT-PCR master mix and 0.25 μl Quantitect RT-mix. The one-step qRT–PCR conditions were 50 °C for 30 min, 95 °C for 15 min followed by 40 cycles of 15 s at 94 °C, 30 s at 60 °C and 30 s at 72 °C with data collection at the third step of each cycle. The amplification specificity was checked by the melting profile of the amplicon and 2% agarose gel electrophoresis. The Ct values were then extracted using the Applied Biosystems 7300 software version 1.3.1. The Ct values of *TST*, *SQRDL* or *COX4* were normalized to the Ct values of *hGAPDH* generating ΔCt. Next, ΔΔCt was calculated by subtracting the average ΔCt of the control group from the ΔCt of each sample. The relative quantification was then calculated as 2^*−*ΔΔCt^ as described previously^66^.

### Microbiota–mitochondrial proteins correlation analysis

Pairwise correlations between the relative abundance of the significant OTUs and mitochondrial proteins identified via MS were calculated using the non-parametric Kendall Tau and Spearman's rank correlation coefficients. To avoid spurious correlations, OTUs and mitochondrial proteins absent in more than half of the samples in each group were excluded from each pairwise comparison. As a result, pairwise correlations were calculated between 62 OTUs and 106 mitochondrial proteins from the 21 patients for which we had both data sets. To further avoid spurious correlations, correlations were considered significant only if the upper-tail probability occurrence was below 0.05 for both the Kendall Tau and the Spearman's rank statistics.

### *Il10*^*−/−*^ mouse experiments and tissue processing

Germ-free SvEv129/C57BL6 *Il10*^*−/−*^;*NF-κB*^*EGFP*^ male/female mice (8–12 weeks old, *n*=12) were transferred to specific pathogen free (SPF) conditions and after 2 weeks mice from a randomly selected cohort (*n*=6) were gavaged once weekly with *A*. *parvulum* (1 × 10^8^ CFUs) for 6 weeks and sacrificed 6 weeks later (14 weeks total). *A. parvulum* ATCC 33793 was grown in fastidious anaerobic broth (Lab M, Canada). To investigate the involvement of complex microbiota in the development of colitis, we performed two subsequent experiments using C57BL6 *Il10*^*−/−*^ male/female mice. In the first experimental setting, gnotobiotic *Il10*^*−/−*^ mice (*n*=16) were randomized into two groups; 1: GF only (*n*=6) and 2: GF+*A*. *parvulum* (1 × 10^8^ CFUs; *n*=10). The mice were killed after 8 weeks of mono-association. The mice were fed normal diet (Teklan Global 18% Protein Rodent Diet). In the second experimental setting, gnotobiotic *Il10*^*−*/*−*^ male/female mice (*n*=31) were transferred to SPF conditions for 2 weeks and then randomized into two groups; 1: SPF only (*n*=7), 2: SPF plus *A*. *parvulum* (1 × 10^8^ CFUs; *n*=8), 3: SPF plus bismuth (III) diet (*n*=8) and 4: SPF plus *A. parvulum* and bismuth (III) diet (*n*=8). As described for the first cohort, the mice were gavaged weekly with *A*. *parvulum* (1 × 10^8^ CFUs) for 6 weeks and then killed after 6 weeks of colonization (14 weeks total). Bismuth (III) subsalicylate (Sigma-Aldrich, Saint Louis, MO, USA) was incorporated to the chow (Teklan Global 18% Protein Rodent Diet) at a concentration of 7 g kg^−1^ (Harlan Laboratories, Madison, WI, USA) and then irradiated for gnotobiotic experiments. The mice were fed with this diet 1 week before the colonization with *A*. *parvulum*. The tissue samples from the colon were collected for RNA and histology as previously described^67^. All the animal procedures were approved by the University of North Carolina at Chapel Hill Animal Care and Use Committee. The histological images were acquired using a DFC310 FX (LEICA) coupled to LEICA Application Suite AFv4.6 software. Intestinal inflammation was blindly scored as previously described^18^. The tissue was divided into four quarters to allow individual scoring of the mid-section, which was affected by *A*. *parvulum* more so than in other colitis models. A score was given to each quarter separately and then added to generate a final colitis score on a scale of 0–16.

### Statistical analyses of *Il10*^*−/−*^ mice results

Unless specifically noted, the statistical analyses were performed using GraphPad Prism version 6 (GraphPad, La Jolla, CA, USA). The comparisons of mouse studies were made with a nonparametric analysis of variance, and then a Mann–Whitney *U-*test. The experiments were considered statistically significant if *P*<0.05.

### Mouse endoscopy

Colonoscopy was performed using a ‘Coloview System' (Karl Storz Veterinary Endoscopy) as described previously^68^. The mice were anaesthetized using 1.5 to 2% isoflurane and ∼4 cm of the colon from the anal verge from the splenic flexure was visualized. The procedures were digitally recorded on an AIDA Compaq PC.

### qRT–PCR on mouse intestinal samples

Total RNA from intestinal tissues was extracted from the distal part of the colon using TRIzol (Invitrogen, Carlsbad, CA, USA) following the manufacturer's protocol. The complementary DNA was reverse-transcribed using M-MLV (Invitrogen, Carlsbad, CA, USA) and mRNA expression levels were measured using SYBR Green PCR Master mix (Applied Biosystems) on an ABI 7900HT Fast Real-Time PCR System and normalized to β-actin. The primers used are listed in Supplementary Table 5. The PCR reactions were performed for 40 cycles according to the manufacturer's recommendations, and RNA fold changes were calculated using the ΔΔCt method^66^.

### Data availability

The demultiplexed reads for all V6-16S experiments that support the findings of this study have been deposited to the NCBI Sequence Read Archive (http://www.ncbi.nlm.nih.gov/sra). The pure culture experiments were submitted under accession number SRP075131, the Illumina microbiota reads under accession numbers SRP034595/SRP056939, the 454 microbiota reads under accession number SRP034632 and the Ion Torrent reads under accession number SRP075131. All the raw data files for the mass spectrometry that support the findings of this study have been submitted to ProteomeXchange under accession number PXD002882. The remaining data are available within the Article and Supplementary Information files or available from the author on request.

## Additional information

**How to cite this article:** Mottawea, W. *et al*. Altered intestinal microbiota–host mitochondria crosstalk in new onset Crohn's disease. *Nat. Commun.* **7,** 13419 doi: 10.1038/ncomms13419 (2016).

**Publisher's note**: Springer Nature remains neutral with regard to jurisdictional claims in published maps and institutional affiliations.

## Supplementary Material

Supplementary Information Supplementary Figures 1-16, Supplementary Tables 1-5 and Supplementary References

Supplementary Data 1 OTUs that differ significantly in abundance in at least one of the two pairwise comparisons performed (controls vs. CD or controls vs. UC; p values calculated using metagenomeSeq).

Supplementary Data 2 Taxa that differ significantly in control vs CD or UC patients by LEfSe analysis.

Supplementary Data 3 Comparison between differentially abundant bacteria using V4/V6 regions and Illumina/Ion Torrent sequencing platforms.

Supplementary Data 4 OTUs that differ significantly in abundance as a function of inflammation severity in CD patients in at least one of the three pairwise comparisons performed (mild vs. moderate; mild vs. severe; and moderate vs. severe).

Supplementary Data 5 Relative abundances and error rates for pure cultures sequenced using the 16S-V6-rDNA protocol.

## Figures and Tables

**Table 1:** Subjects characteristics.

	**Control**	**Crohn's disease**		**Ulcerative colitis**	
Subjects	63	94		37	
Gender (F/M)	33/30	34/60		18/19	
Median age, y (IQ range)	14 (10–16)	14 (11.75–15)		15 (12.5–17)	
Disease activity (Inactive/mild/moderate/severe)*	NA	3/19/25/47		1/4/16/16	
Inflammation location†	NA	Upper GIT	1	E1	2
		Ileum	28	E2	2
		Colon	18	E3	5
		Ileum–colon	47	E4	28

^*^Estimated based on PCDAI/PUCAI; Pediatric CD/UC Activity Index.

^†^E1, ulcerative proctitis; E2, left-sided UC; E3, extensive UC; and E4, pancolitis.

## References

[b1] Rigoli L. & Caruso R. A. Inflammatory bowel disease in pediatric and adolescent patients: a biomolecular and histopathological review. World J. Gastroenterol. 20, 10262–10278 (2014). 25132743 10.3748/wjg.v20.i30.10262 PMC4130834

[b2] Li J., Butcher J., Mack D. & Stintzi A. Functional impacts of the intestinal microbiome in the pathogenesis of inflammatory bowel disease. Inflamm. Bowel Dis. 21, 139–153 (2015). 25248007 10.1097/MIB.0000000000000215

[b3] Gevers D. . The treatment-naive microbiome in new-onset Crohn's disease. Cell Host Microbe 15, 382–392 (2014). 24629344 10.1016/j.chom.2014.02.005 PMC4059512

[b4] Hansen R. . Microbiota of *de novo* pediatric IBD: increased *Faecalibacterium prausnitzii* and reduced bacterial diversity in Crohn's but not in ulcerative colitis. Am. J. Gastroenterol. 107, 1913–1922 (2012). 23044767 10.1038/ajg.2012.335

[b5] Kolho K. L. . Fecal microbiota in pediatric inflammatory bowel disease and its relation to inflammation. Am. J. Gastroenterol. 110, 921–930 (2015). 25986361 10.1038/ajg.2015.149

[b6] Morgan X. C. . Dysfunction of the intestinal microbiome in inflammatory bowel disease and treatment. Genome Biol. 13, R79 (2012). 23013615 10.1186/gb-2012-13-9-r79 PMC3506950

[b7] Papa E. . Non-invasive mapping of the gastrointestinal microbiota identifies children with inflammatory bowel disease. PLoS ONE 7, e39242 (2012). 22768065 10.1371/journal.pone.0039242 PMC3387146

[b8] Presley L. L. . Host-microbe relationships in inflammatory bowel disease detected by bacterial and metaproteomic analysis of the mucosal-luminal interface. Inflamm. Bowel Dis. 18, 409–417 (2012). 21698720 10.1002/ibd.21793 PMC3179764

[b9] McHardy I. H. . Integrative analysis of the microbiome and metabolome of the human intestinal mucosal surface reveals exquisite inter-relationships. Microbiome 1, 17 (2013). 24450808 10.1186/2049-2618-1-17 PMC3971612

[b10] Walters W. A., Xu Z. & Knight R. Meta-analyses of human gut microbes associated with obesity and IBD. FEBS Lett. 588, 4223–4233 (2014). 25307765 10.1016/j.febslet.2014.09.039 PMC5050012

[b11] Segata N. . Metagenomic biomarker discovery and explanation. Genome Biol. 12, R60 (2011). 21702898 10.1186/gb-2011-12-6-r60 PMC3218848

[b12] Paulson J. N., Stine O. C., Bravo H. C. & Pop M. Differential abundance analysis for microbial marker-gene surveys. Nat. Methods 10, 1200–1202 (2013). 24076764 10.1038/nmeth.2658 PMC4010126

[b13] Salter S. J. . Reagent and laboratory contamination can critically impact sequence-based microbiome analyses. BMC Biol. 12, 87 (2014). 25387460 10.1186/s12915-014-0087-z PMC4228153

[b14] Schloss P. D., Gevers D. & Westcott S. L. Reducing the effects of PCR amplification and sequencing artifacts on 16S rRNA-based studies. PLoS ONE 6, e27310 (2011). 22194782 10.1371/journal.pone.0027310 PMC3237409

[b15] Carbonero F., Benefiel A. C., Alizadeh-Ghamsari A. H. & Gaskins H. R. Microbial pathways in colonic sulfur metabolism and links with health and disease. Front. Physiol. 3, 448 (2012). 23226130 10.3389/fphys.2012.00448 PMC3508456

[b16] Tyanova S. . Proteomic maps of breast cancer subtypes. Nat. Commun. 7, 10259 (2016). 26725330 10.1038/ncomms10259 PMC4725767

[b17] Blachier F. . Luminal sulfide and large intestine mucosa: friend or foe? Amino Acids 39, 335–347 (2010). 20020161 10.1007/s00726-009-0445-2

[b18] Karrasch T., Kim J. S., Muhlbauer M., Magness S. T. & Jobin C. Gnotobiotic IL-10^*−*/*−*^;NF-kappa B(EGFP) mice reveal the critical role of TLR/NF-kappa B signaling in commensal bacteria-induced colitis. J. Immunol. 178, 6522–6532 (2007). 17475882 10.4049/jimmunol.178.10.6522

[b19] Kim S. C., Tonkonogy S. L., Karrasch T., Jobin C. & Sartor R. B. Dual-association of gnotobiotic IL-10^*−*/*−*^ mice with 2 nonpathogenic commensal bacteria induces aggressive pancolitis. Inflamm. Bowel Dis. 13, 1457–1466 (2007). 17763473 10.1002/ibd.20246

[b20] Suarez F. L., Furne J. K., Springfield J. & Levitt M. D. Bismuth subsalicylate markedly decreases hydrogen sulfide release in the human colon. Gastroenterology 114, 923–929 (1998). 9558280 10.1016/s0016-5085(98)70311-7

[b21] Sifroni K. G. . Mitochondrial respiratory chain in the colonic mucosal of patients with ulcerative colitis. Mol. Cell. Biochem. 342, 111–115 (2010). 20440543 10.1007/s11010-010-0474-x

[b22] O'Morain C., Smethurst P., Levi J. & Peters T. J. Subcellular fractionation of rectal biopsy homogenates from patients with inflammatory bowel disease. Scand. J. Gastroenterol. 20, 209–214 (1985). 3992179 10.3109/00365528509089659

[b23] Soderholm J. D. . Augmented increase in tight junction permeability by luminal stimuli in the non-inflamed ileum of Crohn's disease. Gut 50, 307–313 (2002). 11839706 10.1136/gut.50.3.307 PMC1773145

[b24] Beltran B. . Mitochondrial dysfunction, persistent oxidative damage, and catalase inhibition in immune cells of naive and treated Crohn's disease. Inflamm. Bowel Dis. 16, 76–86 (2010). 19637347 10.1002/ibd.21027

[b25] Restivo N. L., Srivastava M. D., Schafer I. A. & Hoppel C. L. Mitochondrial dysfunction in a patient with crohn disease: possible role in pathogenesis. J. Pediatr. Gastroenterol. Nutr. 38, 534–538 (2004). 15097444 10.1097/00005176-200405000-00014

[b26] Bar F. . Mitochondrial gene polymorphisms that protect mice from colitis. Gastroenterology 145, 1055–1063 (2013). 23872498 10.1053/j.gastro.2013.07.015

[b27] Wang A. . Targeting mitochondria-derived reactive oxygen species to reduce epithelial barrier dysfunction and colitis. Am. J. Pathol. 184, 2516–2527 (2014). 25034594 10.1016/j.ajpath.2014.05.019 PMC4188172

[b28] Soderholm J. D. . Epithelial permeability to proteins in the noninflamed ileum of Crohn's disease? Gastroenterology 117, 65–72 (1999). 10381911 10.1016/s0016-5085(99)70551-2

[b29] Zeissig S. . Changes in expression and distribution of claudin 2, 5 and 8 lead to discontinuous tight junctions and barrier dysfunction in active Crohn's disease. Gut 56, 61–72 (2007). 16822808 10.1136/gut.2006.094375 PMC1856677

[b30] Ramasamy S., Singh S., Taniere P., Langman M. J. & Eggo M. C. Sulfide-detoxifying enzymes in the human colon are decreased in cancer and upregulated in differentiation. Am. J. Physiol. Gastrointest. Liver Physiol. 291, G288–G296 (2006). 16500920 10.1152/ajpgi.00324.2005

[b31] Linden D. R. Hydrogen sulfide signaling in the gastrointestinal tract. Antioxid. Redox Signal. 20, 818–830 (2014). 23582008 10.1089/ars.2013.5312 PMC3910452

[b32] Quince C. . Extensive modulation of the fecal metagenome in children with Crohn's disease during exclusive enteral nutrition. Am. J. Gastroenterol. 110, 1718–1729 (2015). 26526081 10.1038/ajg.2015.357 PMC4697132

[b33] Miller T. W. . Hydrogen sulfide is an endogenous potentiator of T cell activation. J. Biol. Chem. 287, 4211–4221 (2012). 22167178 10.1074/jbc.M111.307819 PMC3281711

[b34] Ijssennagger N. . Gut microbiota facilitates dietary heme-induced epithelial hyperproliferation by opening the mucus barrier in colon. Proc. Natl Acad. Sci. USA 112, 10038–10043 (2015). 26216954 10.1073/pnas.1507645112 PMC4538683

[b35] Guo F. F., Yu T. C., Hong J. & Fang J. Y. Emerging roles of hydrogen sulfide in inflammatory and neoplastic colonic diseases. Front. Physiol. 7, 156 (2016). 27199771 10.3389/fphys.2016.00156 PMC4853395

[b36] Wang R. Physiological implications of hydrogen sulfide: a whiff exploration that blossomed. Physiol. Rev. 92, 791–896 (2012). 22535897 10.1152/physrev.00017.2011

[b37] Gerasimidis K. . Decline in presumptively protective gut bacterial species and metabolites are paradoxically associated with disease improvement in pediatric Crohn's disease during enteral nutrition. Inflamm. Bowel Dis. 20, 861–871 (2014). 24651582 10.1097/MIB.0000000000000023

[b38] Ungaro R. . Antibiotics associated with increased risk of new-onset Crohn's disease but not ulcerative colitis: a meta-analysis. Am. J. Gastroenterol. 109, 1728–1738 (2014). 25223575 10.1038/ajg.2014.246

[b39] North American Society for Pediatric Gastroenterology, Hepatology and Nutrition. . Differentiating ulcerative colitis from Crohn disease in children and young adults: report of a working group of the North American Society for Pediatric Gastroenterology, Hepatology, and Nutrition and the Crohn's and Colitis Foundation of America. J. Pediatr. Gastroenterol. Nutr. 44, 653–674 (2007). 17460505 10.1097/MPG.0b013e31805563f3

[b40] Levine A. . Pediatric modification of the Montreal classification for inflammatory bowel disease: the Paris classification. Inflamm. Bowel Dis. 17, 1314–1321 (2011). 21560194 10.1002/ibd.21493

[b41] Hyams J. . Evaluation of the pediatric crohn disease activity index: a prospective multicenter experience. J. Pediatr. Gastroenterol. Nutr. 41, 416–421 (2005). 16205508 10.1097/01.mpg.0000183350.46795.42

[b42] Turner D. . Development, validation, and evaluation of a pediatric ulcerative colitis activity index: a prospective multicenter study. Gastroenterology 133, 423–432 (2007). 17681163 10.1053/j.gastro.2007.05.029

[b43] Harris P. A. . Research electronic data capture (REDCap)—a metadata-driven methodology and workflow process for providing translational research informatics support. J. Biomed. Inform. 42, 377–381 (2009). 18929686 10.1016/j.jbi.2008.08.010 PMC2700030

[b44] Jimenez-Rivera C., Haas D., Boland M., Barkey J. L. & Mack D. R. Comparison of two common outpatient preparations for colonoscopy in children and youth. Gastroenterol. Res. Pract. 2009, 518932 (2009). 20029642 10.1155/2009/518932 PMC2796226

[b45] Sundquist A. . Bacterial flora-typing with targeted, chip-based Pyrosequencing. BMC Microbiol. 7, 108 (2007). 18047683 10.1186/1471-2180-7-108 PMC2244631

[b46] Magoc T. & Salzberg S. L. FLASH: fast length adjustment of short reads to improve genome assemblies. Bioinformatics 27, 2957–2963 (2011). 21903629 10.1093/bioinformatics/btr507 PMC3198573

[b47] Caporaso J. G. . QIIME allows analysis of high-throughput community sequencing data. Nat. Methods 7, 335–336 (2010). 20383131 10.1038/nmeth.f.303 PMC3156573

[b48] Letunic I. & Bork P. Interactive Tree Of Life (iTOL): an online tool for phylogenetic tree display and annotation. Bioinformatics 23, 127–128 (2007). 17050570 10.1093/bioinformatics/btl529

[b49] Letunic I. & Bork P. Interactive Tree Of Life v2: online annotation and display of phylogenetic trees made easy. Nucleic Acids Res. 39, W475–W478 (2011). 21470960 10.1093/nar/gkr201 PMC3125724

[b50] McMurdie P. J. & Holmes S. Phyloseq: a bioconductor package for handling and analysis of high-throughput phylogenetic sequence data. Pac. Symp. Biocomput. 235–246 (2012). 22174279 PMC3357092

[b51] Milani C. . Assessing the fecal microbiota: an optimized ion torrent 16S rRNA gene-based analysis protocol. PLoS ONE 8, e68739 (2013). 23869230 10.1371/journal.pone.0068739 PMC3711900

[b52] Li H. . The Sequence Alignment/Map format and SAMtools. Bioinformatics 25, 2078–2079 (2009). 19505943 10.1093/bioinformatics/btp352 PMC2723002

[b53] Aronesty E. ea-utils: ‘Command-line tools for processing biological sequencing data'. https://github.com/ExpressionAnalysis/ea-utils (2011).

[b54] Edgar R. C. Search and clustering orders of magnitude faster than BLAST. Bioinformatics 26, 2460–2461 (2010). 20709691 10.1093/bioinformatics/btq461

[b55] Altschul S. F., Gish W., Miller W., Myers E. W. & Lipman D. J. Basic local alignment search tool. J. Mol. Biol. 215, 403–410 (1990). 2231712 10.1016/S0022-2836(05)80360-2

[b56] Cole J. R. . The Ribosomal Database Project: improved alignments and new tools for rRNA analysis. Nucleic Acids Res. 37, D141–D145 (2009). 19004872 10.1093/nar/gkn879 PMC2686447

[b57] Hippe B. . Quantification of butyryl CoA:acetate CoA-transferase genes reveals different butyrate production capacity in individuals according to diet and age. FEMS Microbiol. Lett. 316, 130–135 (2011). 21204931 10.1111/j.1574-6968.2010.02197.x

[b58] Louis P. & Flint H. J. Development of a semiquantitative degenerate real-time pcr-based assay for estimation of numbers of butyryl-coenzyme A (CoA) CoA transferase genes in complex bacterial samples. Appl. Environ. Microbiol. 73, 2009–2012 (2007). 17259367 10.1128/AEM.02561-06 PMC1828812

[b59] Walker A. W. . Dominant and diet-responsive groups of bacteria within the human colonic microbiota. ISME J. 5, 220–230 (2011). 20686513 10.1038/ismej.2010.118 PMC3105703

[b60] Faust K. . Microbial co-occurrence relationships in the human microbiome. PLoS Comput. Biol. 8, e1002606 (2012). 22807668 10.1371/journal.pcbi.1002606 PMC3395616

[b61] Shannon P. . Cytoscape: a software environment for integrated models of biomolecular interaction networks. Genome Res. 13, 2498–2504 (2003). 14597658 10.1101/gr.1239303 PMC403769

[b62] Wisniewski J. R., Zougman A., Nagaraj N. & Mann M. Universal sample preparation method for proteome analysis. Nat. Methods 6, 359–362 (2009). 19377485 10.1038/nmeth.1322

[b63] Stahl M. . L-fucose utilization provides *Campylobacter jejuni* with a competitive advantage. Proc. Natl Acad. Sci. USA 108, 7194–7199 (2011). 21482772 10.1073/pnas.1014125108 PMC3084102

[b64] Palmer C., Bik E. M., DiGiulio D. B., Relman D. A. & Brown P. O. Development of the human infant intestinal microbiota. PLoS Biol. 5, e177 (2007). 17594176 10.1371/journal.pbio.0050177 PMC1896187

[b65] Ye J. . Primer-BLAST: a tool to design target-specific primers for polymerase chain reaction. BMC Bioinformatics 13, 134 (2012). 22708584 10.1186/1471-2105-13-134 PMC3412702

[b66] Arthur J. C. . Intestinal inflammation targets cancer-inducing activity of the microbiota. Science 338, 120–123 (2012). 22903521 10.1126/science.1224820 PMC3645302

[b67] Sun X., Threadgill D. & Jobin C. *Campylobacter jejuni* induces colitis through activation of mammalian target of rapamycin signaling. Gastroenterology 142, 86–95 (2012). 21963787 10.1053/j.gastro.2011.09.042 PMC3253301

[b68] Lippert E. . Gnotobiotic IL-10; NF-kappaB mice develop rapid and severe colitis following *Campylobacter jejuni* infection. PLoS ONE 4, e7413 (2009). 19841748 10.1371/journal.pone.0007413 PMC2760752

