# Supplementary material for: Altered intestinal microbiota–host mitochondria crosstalk in new onset Crohn's disease
Source: Nat Commun. 2016 Nov 23;7:13419. doi: 10.1038/ncomms13419 (PMC5122959; doi:10.1038/ncomms13419)
Supplement: Supplementary Information — Supplementary Figures 1-16, Supplementary Tables 1-5 and Supplementary References [file ncomms13419-s1.pdf]

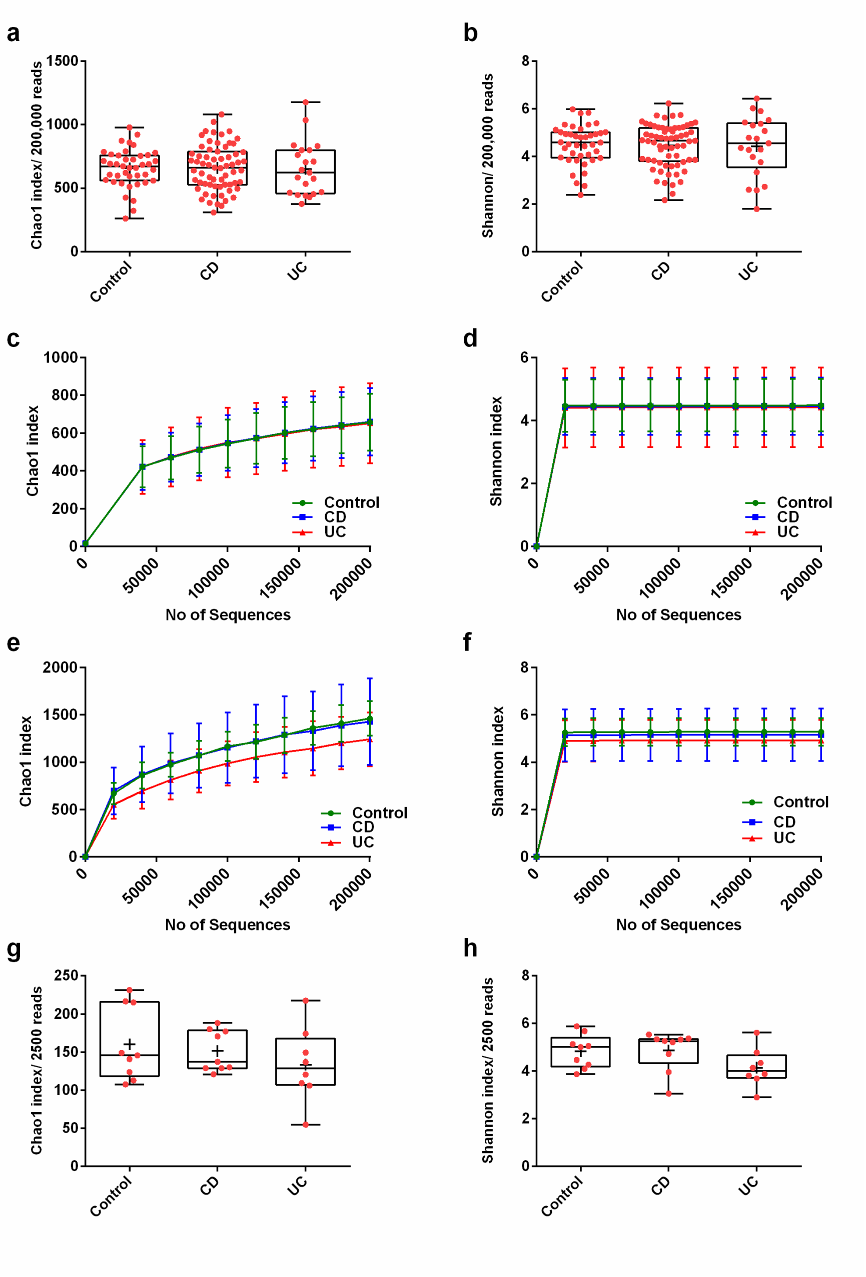

Supplementary Figure 1: The gut microbiota exhibits similar diversity at the right colon of pediatric patients with IBD in comparison to control children. Richness and diversity of the intestinal microbiota were estimated via Chao1 and Shannon indices as calculated by QIIME1.8. **a-b,** Diversity measures of right colon MLI microbiota in control subjects (n= 42) and patients with Crohn's disease (CD; n= 65) and ulcerative colitis (UC; n=21). **c-d,** Diversity measures for the samples as in a-b at different rarefaction depths. **e-f,** Diversity measures of the microbiota from mucosal biopsies collected from control subjects (n=9), CD children (n=20) and UC patients (n=8). **g-h,** diversity measures of MLI microbiota per 2500 pyrosequencing reads in control (n=9), CD (n=9) and UC (n=8) subjects. Statistical comparisons were done by Kruskal-Wallis followed by Dunn's multiple comparisons. All comparisons were non-significant.

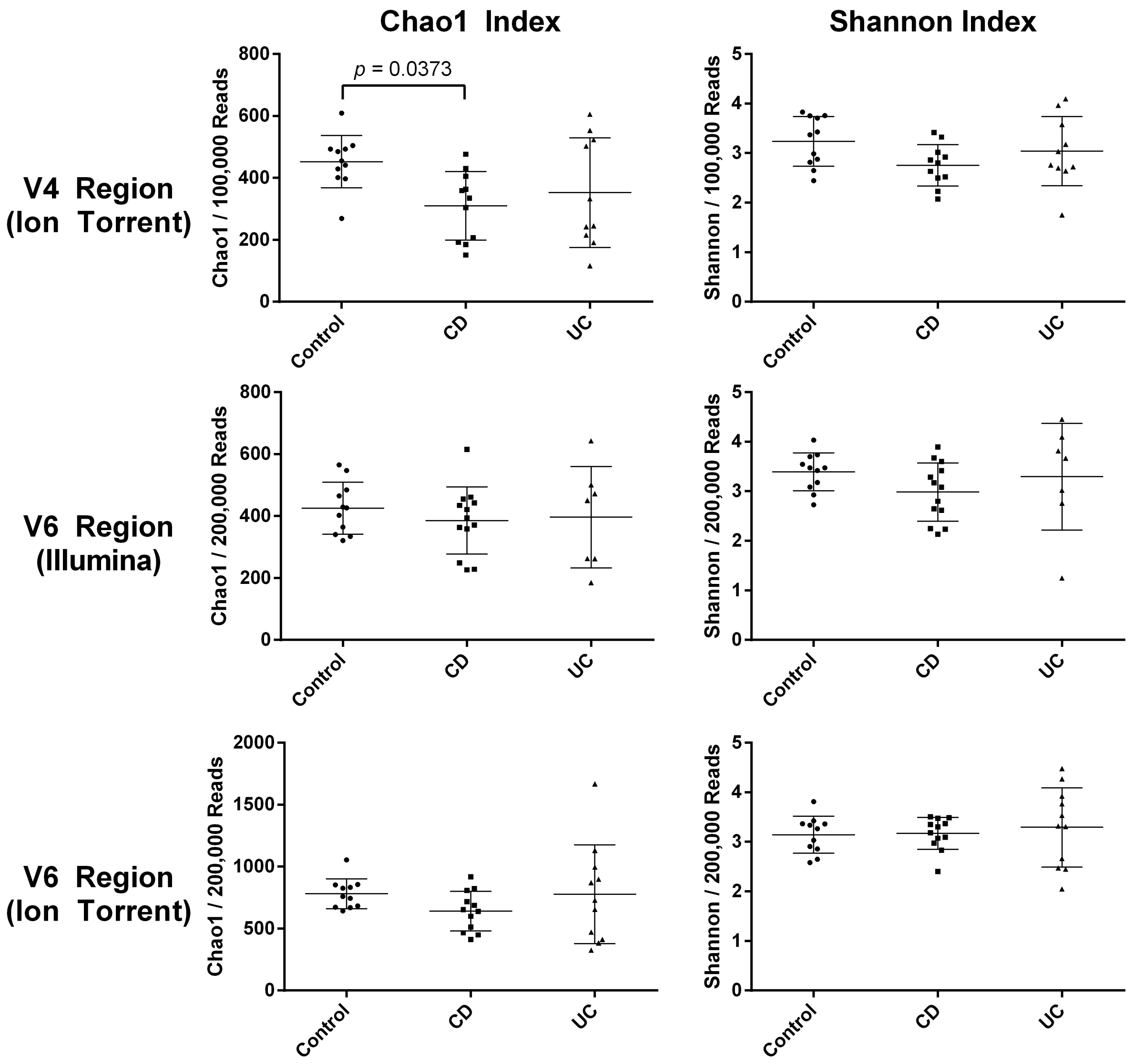

Supplementary Figure 2: Chao1/Shannon indices as measured by V4/V6 sequencing using Illumina/Ion Torrent platforms No differences were observed between control, CD and UC groups in the Shannon index under any sequencing target/platform. V4 sequencing suggests a lower Chao1 index between Control and CD patients, while no difference is seen between Controls and CD when targeting the V6 region. Statistical differences tested using a Kruskal-Wallis non-parametric test with a Dunn's post hoc test. All comparisons were non-significant unless indicated otherwise.

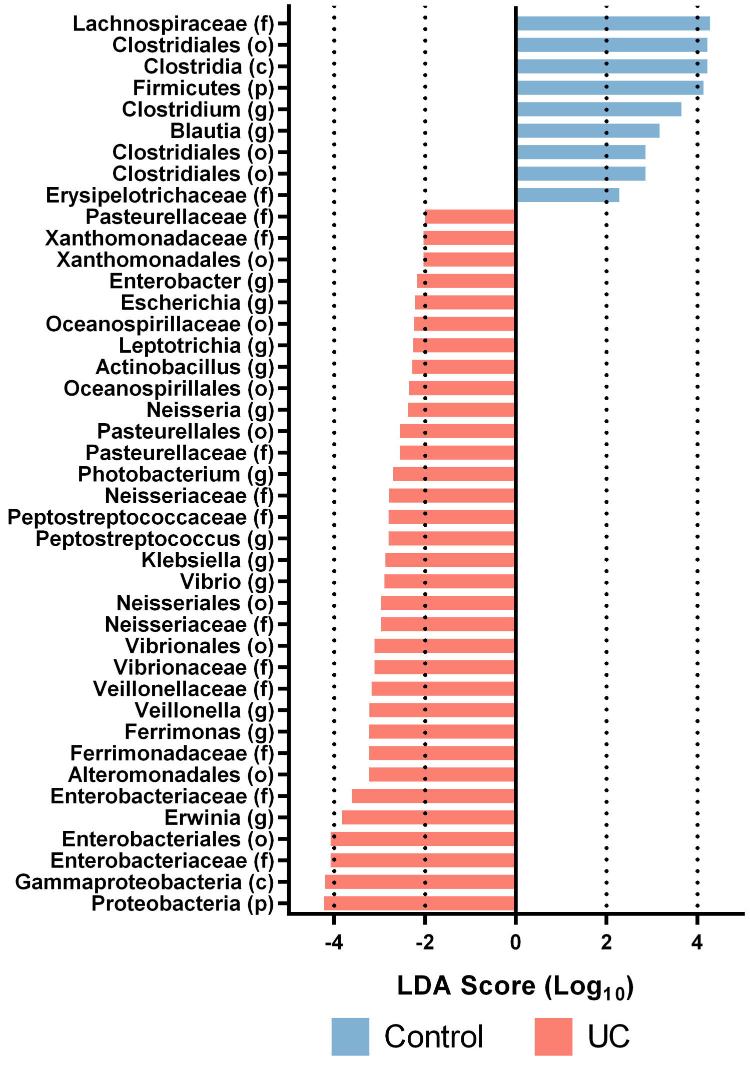

Supplementary Figure 3: Histogram of linear discriminant analysis (LDA) effect size score for UC differentially abundant taxa compared to controls (n=21 and 42 for UC and controls, respectively); only OTUs meeting an LDA significant threshold ≥2 with a $p<0.05$ are shown.

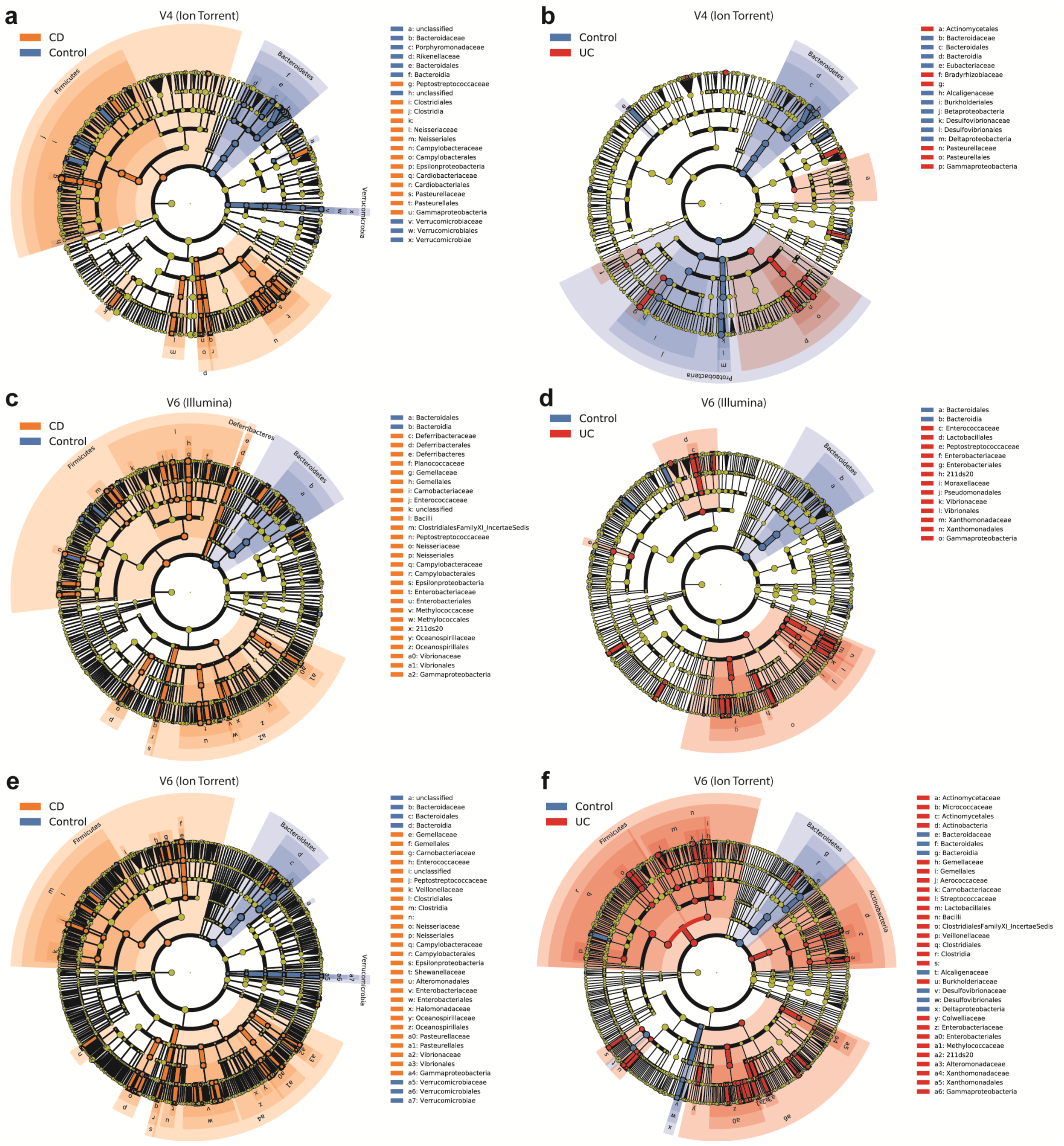

Supplementary Figure 4: V4/V6 sequencing using either Illumina/Ion Torrent platforms identifies similar differentially abundant taxa. Differentially abundant taxa were identified using LEfSe between control/CD (a,c,e) and control/UC (b,d,f) using the results from V4 Ion torrent sequencing (a,b), V6 Illumina sequencing (c,d) and V6 Ion torrent sequencing (e,f). The identified differentially abundant taxa are highly concordant across all three 16S datasets for both CD and UC. See Supplementary Data 3 for more information.

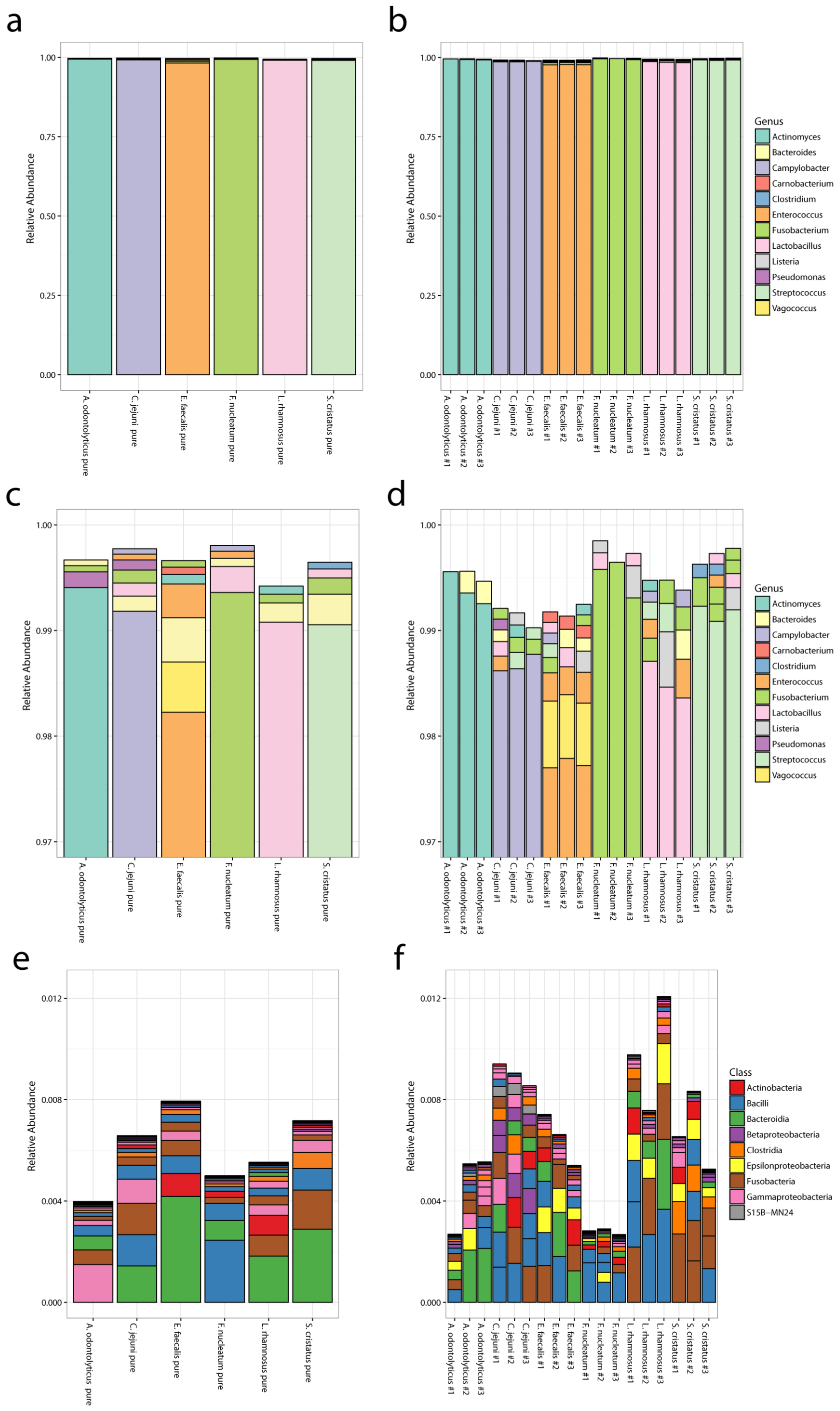

Supplementary Figure 5: 16S-V6-rDNA profiling is robust to sequencing errors and contaminating OTUs: 16S V6-rDNA results from six pure cultures of bacteria extracted directly (a, c, e) or re-extracted (b, d, f) through the FastDNA Spin kit. Relative abundance of the OTUs identified in both experiments are shown (a, b) along with a zoom-in of low abundant OTUs (c, d). Contaminate OTUs (present in >90% of each group) are also listed (e, f). For panels a-d, OTUs with a relative abundance <0.01% were excluded on a sample by sample basis for simplicity while panels e-f display all relevant OTUs regardless of relative abundance.

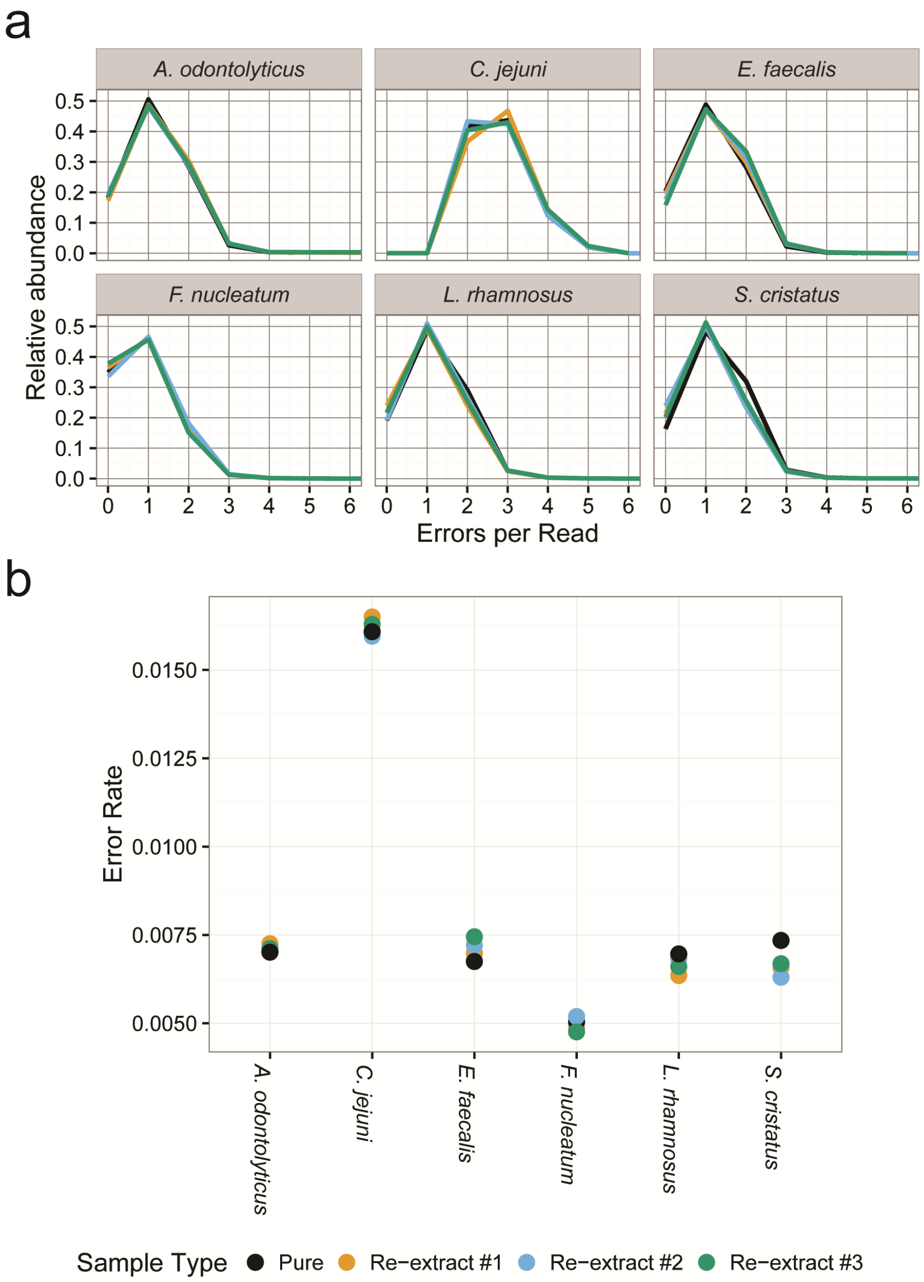

Supplementary Figure 6: The 16S-V6-rDNA amplification protocol has a low error rate: Analyzing the reads for each bacterial V6-16S library preparation revealed that most reads had ≤2 errors present (a). This corresponds to a median error rate of 0.007 (b). Note that *C. jejuni* reads have higher than average errors associated with them due to mismatches within the regions bound by the conserved primers used in the V6 amplification protocol (1 mismatch each in the forward and reverse primer).

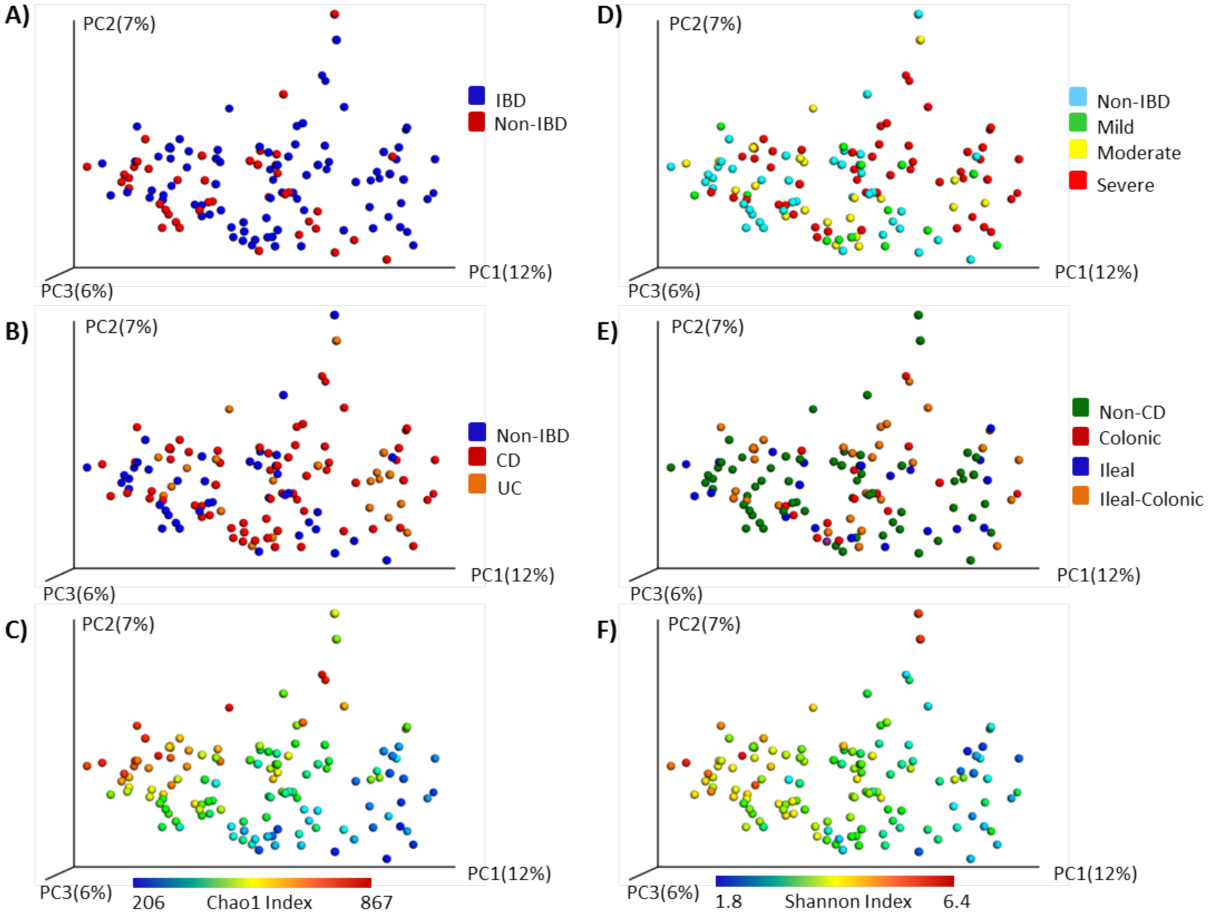

Supplementary Figure 7: Principal Coordinate Analyses (PCoA) based on un-weighted UniFrac distances. PCoA was conducted using QIIME 1.8 and 500,000 randomly selected reads per sample. The samples were colored either by disease status **a,** IBD phenotype **b,** Chao1 estimator **c,** inflammation severity **d,** CD involved location **e,** or Shannon entropy **f**. PC1, PC2 and PC3 represent the top three principal coordinates that captured the highest variability between samples and the percentage shown indicates the fraction of variation captured by each coordinate.

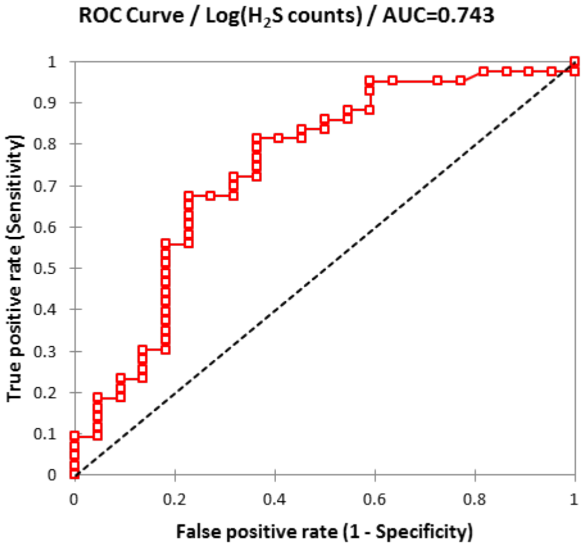

Area under the curve (AUC):

AUC	Standard error	Lower bound (95%)	Upper bound (95%)
0.743	0.067	0.611	0.874

95% confidence interval on the difference between the AUC and 0.5 (Two-tailed test):

Difference	0.243
z (Observed value)	3.607
z (Critical value)	1.960
p-value (Two-tailed)	<0.00031
alpha	0.05

Supplementary Figure 8: Classification of CD patients with severe inflammation based on the relative abundance of H_2S producers. Receiver operating characteristic (ROC) curve for the classification of CD patients with severe inflammation and associated statistics based on the relative abundance of H_2S producers.

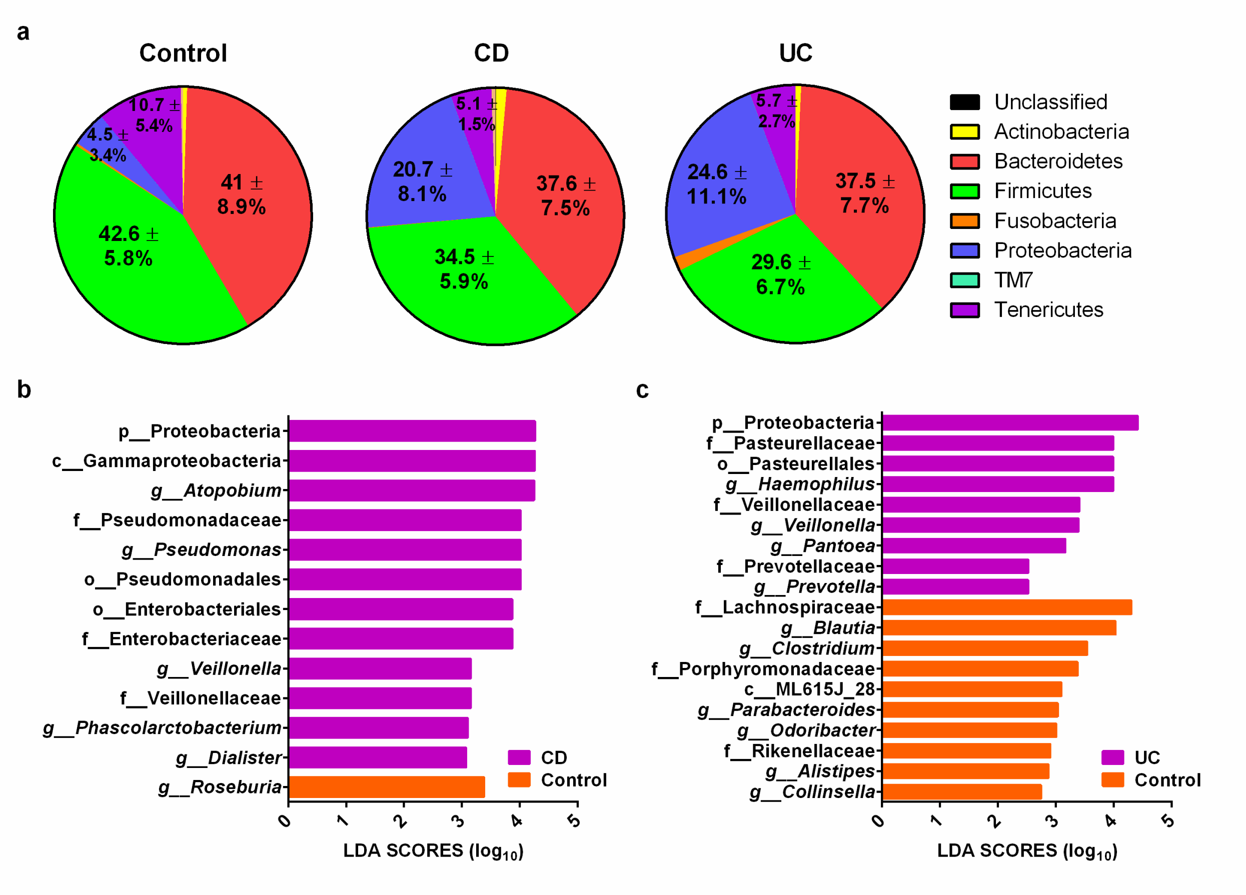

Supplementary Figure 9: Taxonomic composition of the RC microbiota of IBD patients and control subjects based on 16S rRNA pyrosequencing reads. Metagenomic DNA extracted from samples from non-IBD controls (n=9), Crohn's disease (CD; n=9) subjects and ulcerative colitis (UC; n=8) subjects were subjected to 454-pyrosequencing and the generated raw data were analyzed using QIIME 1.8. **a,** Average relative abundance ± SEM of bacterial phyla identified in patients with CD, UC and control subjects. **b,** Histogram of the LDA effect size score for CD-specific differentially abundant taxa. **c,** Histogram of the LDA effect size score for UC-specific differentially abundant taxa.

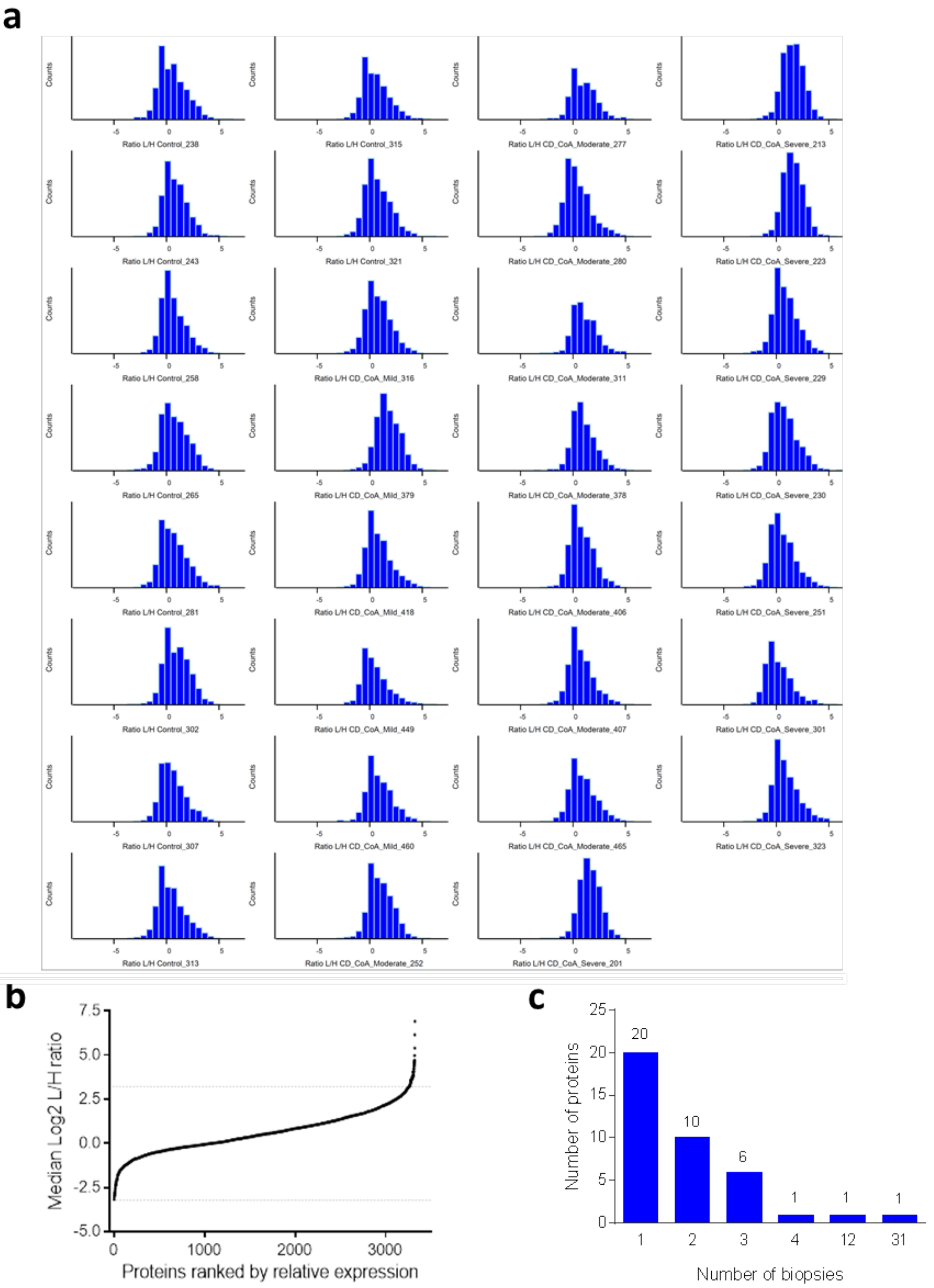

Supplementary Figure 10: Evaluation of super-SILAC for quantitative analysis of colon biopsy proteomes. (a) Histograms indicating the ratio distribution between the individual biopsy proteomes and the super-SILAC standard (non-normalized L/H ratios). (b) Median ratio distribution between individual biopsy proteins and the super-SILAC standard. The dotted lines indicate a 10-fold ratio between the median ratio of patient samples and the super-SILAC standard. (c) Plot of the number of proteins that were identified by light (biopsy) but without a heavy (super-SILAC) counterpart as compared with the number of biopsies from which that protein was identified in.

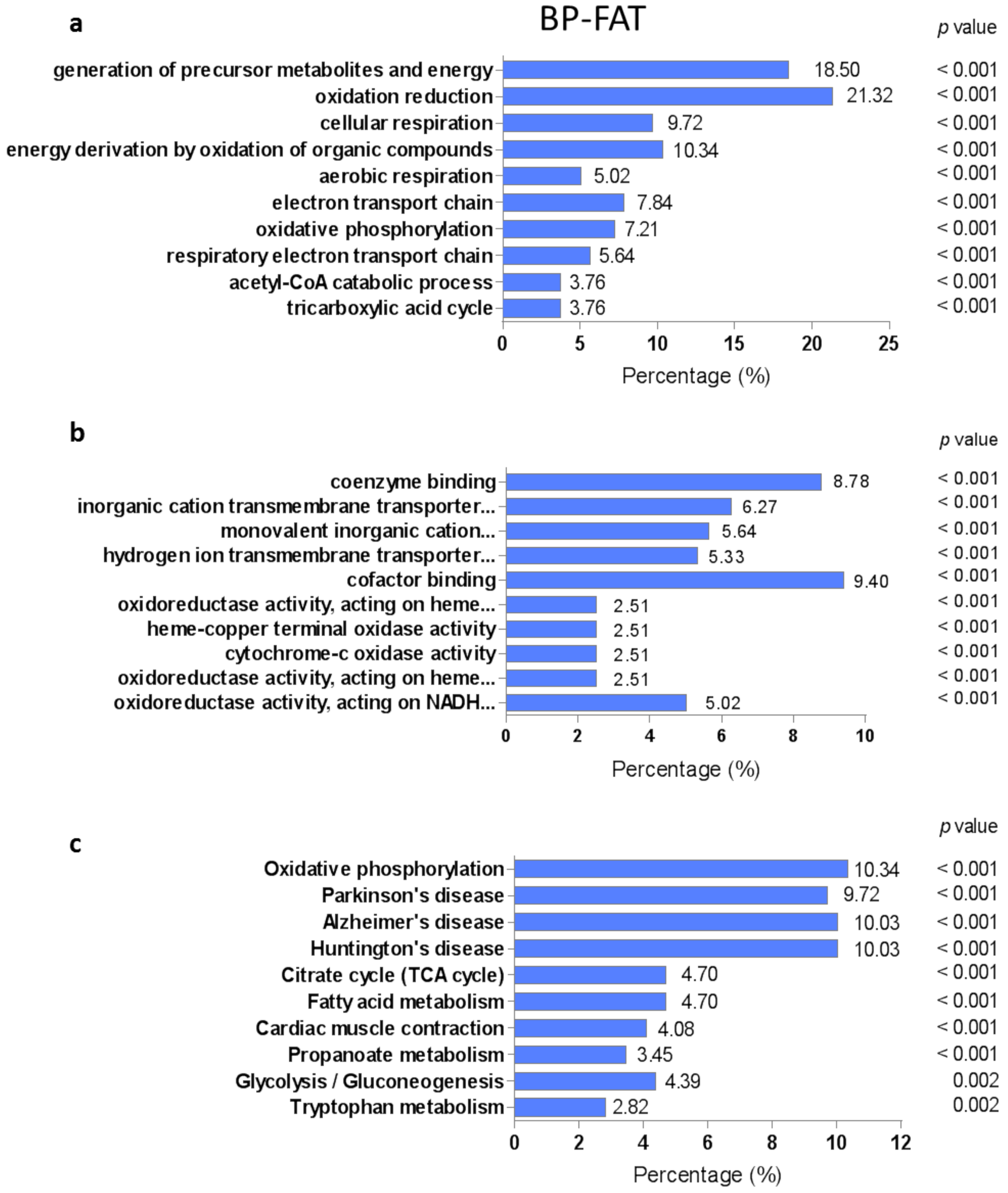

Supplementary Figure 11: Top representative Gene Ontology (GO) and KEGG pathways enriched in CD patients as compared to control subjects. Functional annotation tool (FAT) of the differentially expressed proteins; the 10 most significantly enriched functional groups (GO terms) are shown ($p<10^{-13}$); BP: biological processes (panel **a**); MF: molecular functions (panel **b**) and KEGG pathways (panel **c**)). All classifications were significantly enriched compared to the entire proteomic dataset with $p< 0.05$ (Fisher's exact test).

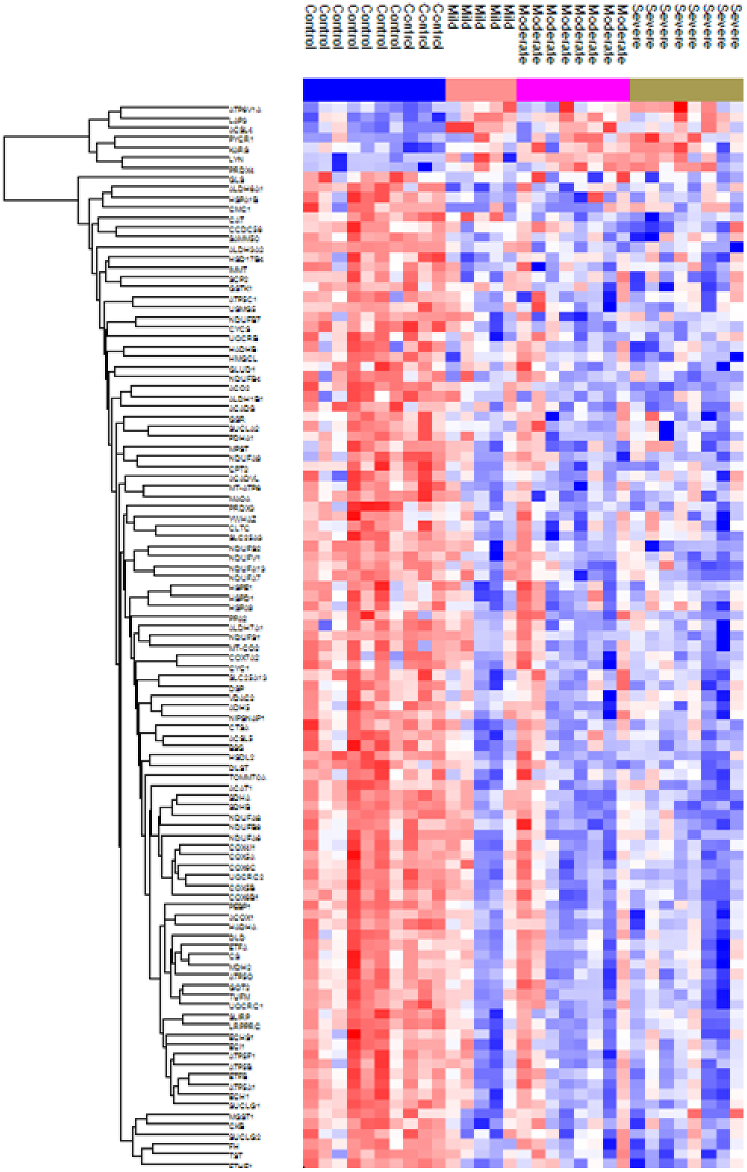

Supplementary Figure 12: Hierarchical clustering of mitochondrial proteins found to be differentially expressed between control subjects and CD patients. The heat map provides a graphical representation of row-normalized expression levels of individual mitochondrial proteins as a function of the disease status (control or CD with mild, moderate or severe inflammation). A color change from red to blue indicates a decrease in protein expression level.

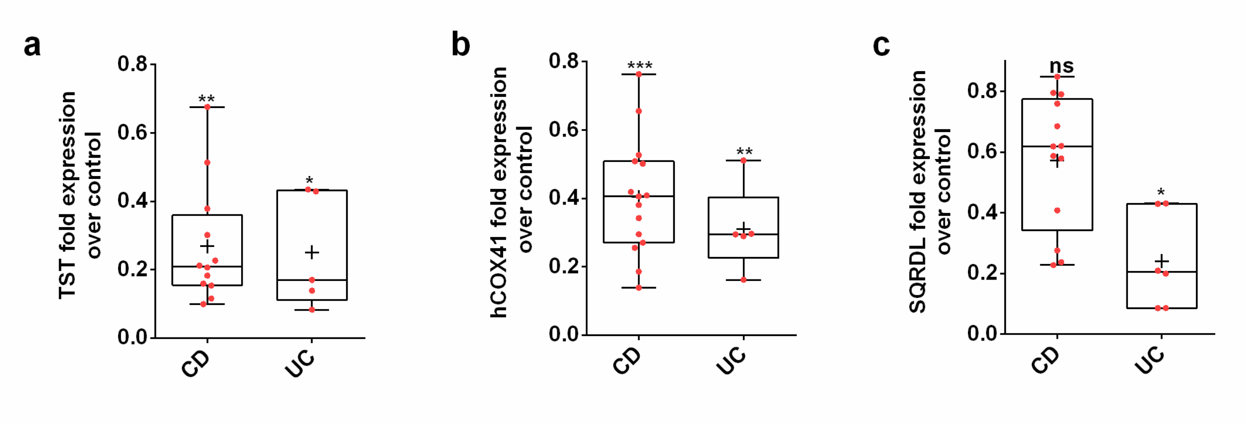

Supplementary Figure 13: Expression level of mitochondrial sulfide detoxification pathway and cytochrome c oxidase is down regulated in IBD. qRT-PCR of thiosulfate sulfur transferase (TST; **a**); cytochrome c oxidase subunit IV (hcox41; **b**); sulfide dehydrogenase (SQR; **c**); normalized to control; n=5 to 6 for UC, 11 to 13 for CD and 10 to 15 for controls. Statistical significance was assessed using a two-tailed Mann-Whitney test (ns; not significant, *p <0.05, **p < 0.01 and ***p < 0.001).

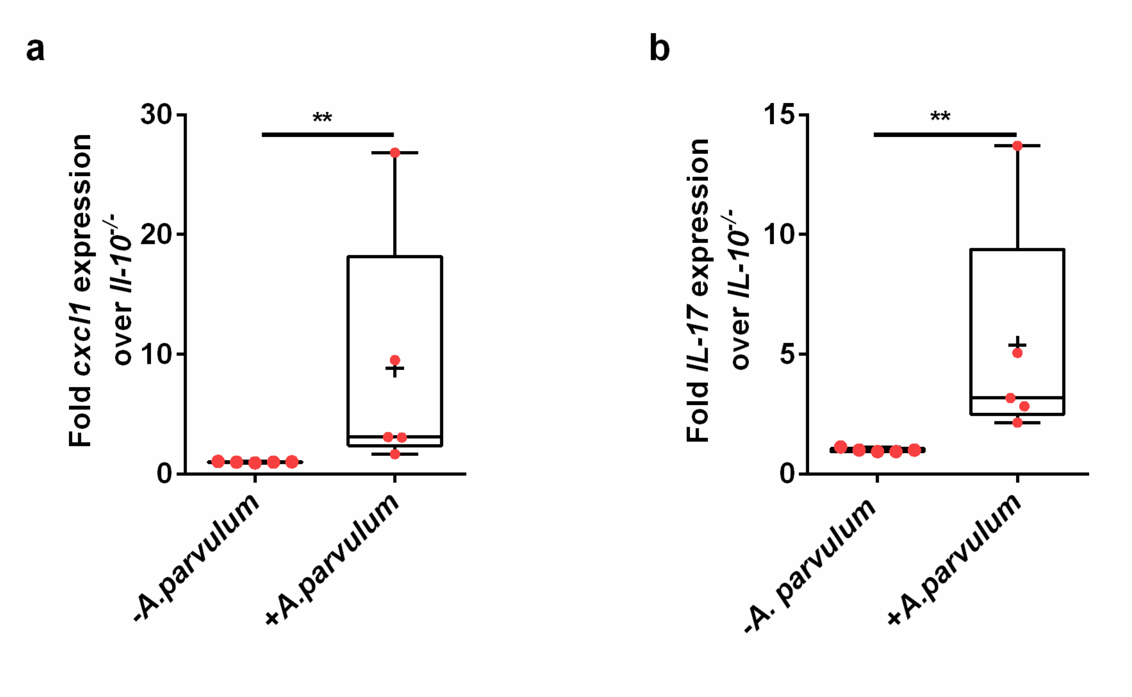

Supplementary Figure 14: *A. parvulum* induces cytokine expression in conventionalized *Il10*$^{-/-}$ mice. Germ-free *Il10*$^{-/-}$ mice were gavaged or not with *A. parvulum* and kept under SPF conditions (n=5 per group). Total RNA was extracted from colonic intestinal tissues 6 weeks post-gavage and *Cxcl1* and *Il-17* expression was measured by qRT-PCR, panels **a** and **b**. Data is expressed relative to the mean of the –*A. parvulum* control group. Crosses indicate mean (+) and the middle line indicates median. A Mann-Whitney U test was performed to assess statistical significance (** p<0.01).

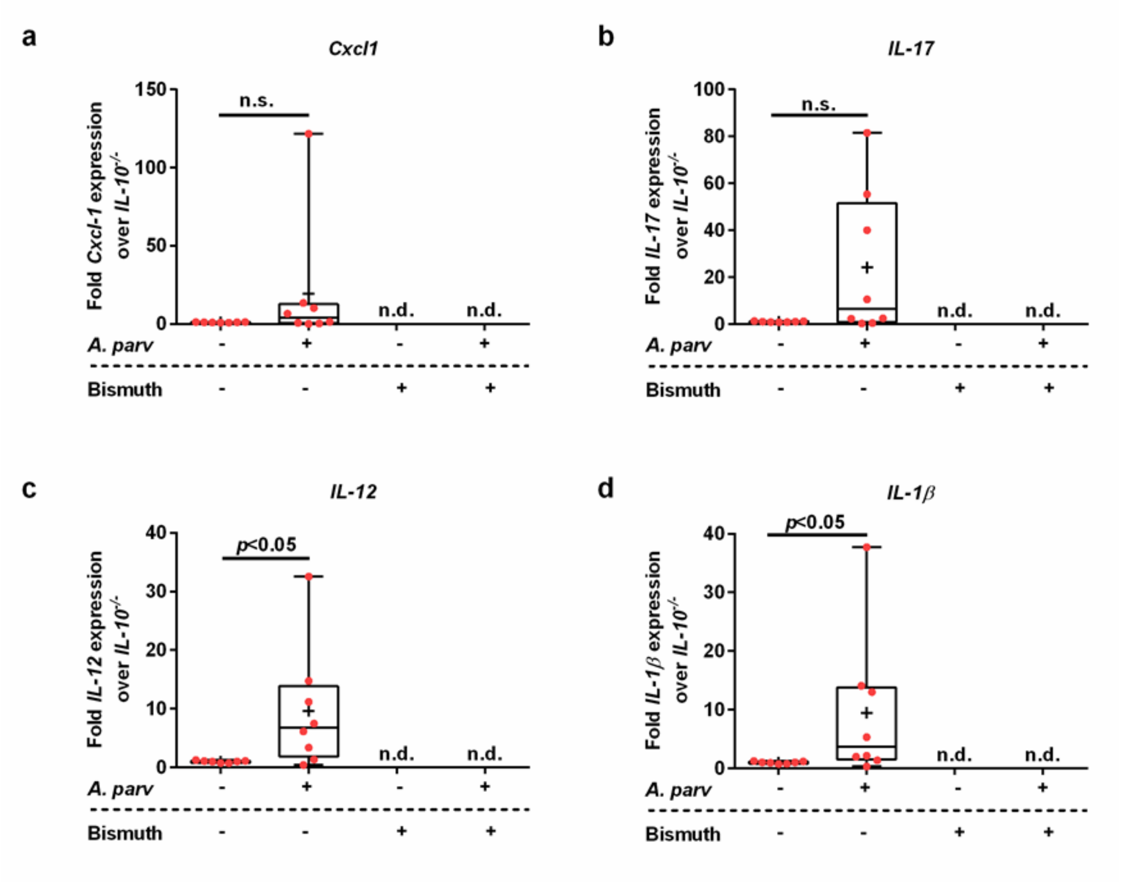

Supplementary Figure 15: *A. parvulum* induces cytokine expression in conventionalized *Il-10*$^{-/-}$ mice. C57Bl6, *Il-10*$^{-/-}$ mice were associated or not with *A. parvulum* and treated or not with bismuth and kept under SPF conditions (n=7-8 per group). Total RNA was extracted from colonic intestinal tissues 6 weeks post-association and *Cxcl1, Il-17, Il-12, and Il-1β* expressions (a-d) were measured by qRT-PCR. Crosses indicate mean and the middle line indicates median (n.d.; not detected). A Mann-Whitney U test was performed to assess statistical significance (n.s.; non-significant).

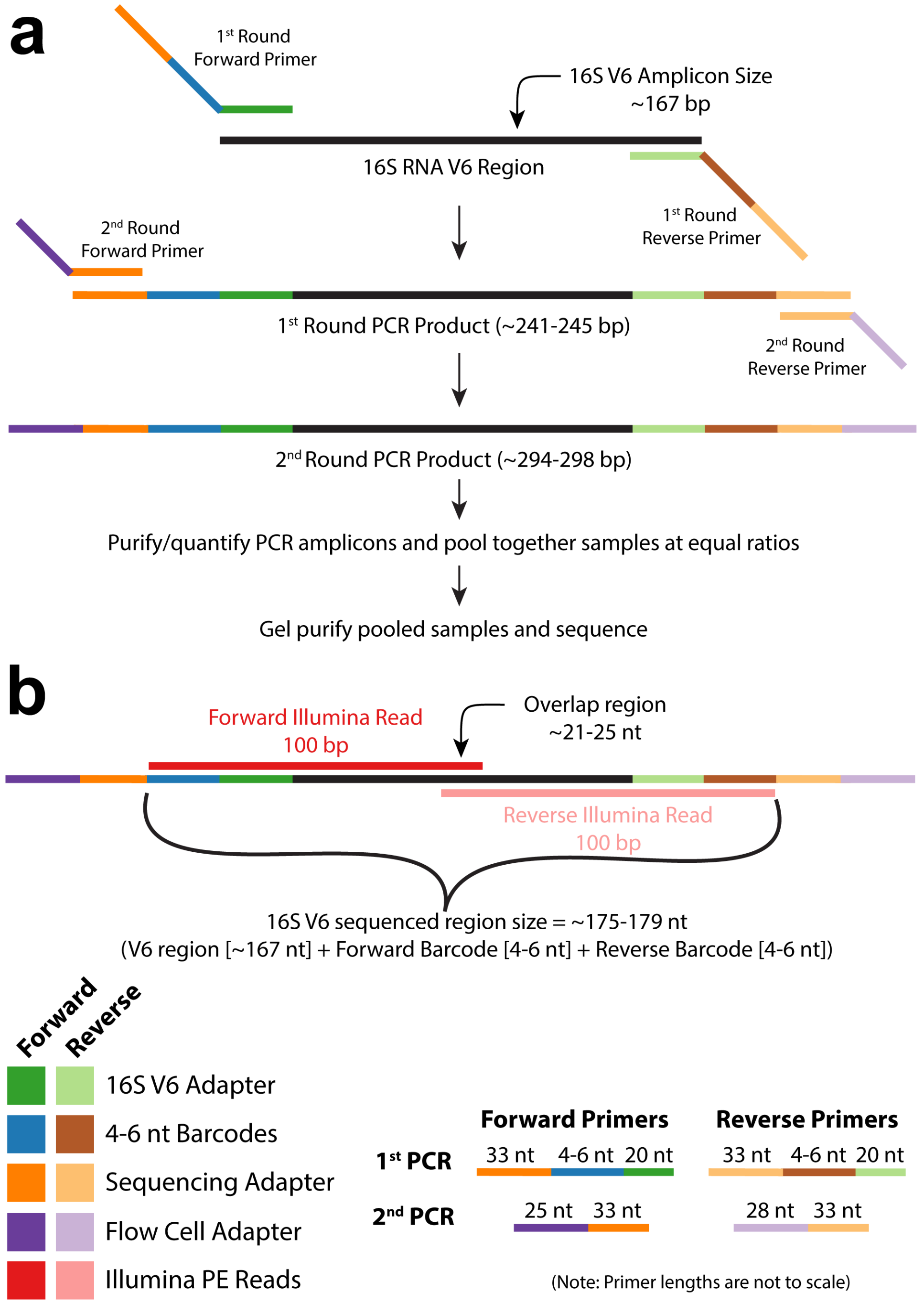

Supplementary Figure 16: 16S rRNA –V6 library construction for Illumina sequencing. (A) Two successive PCR reactions are used to amplify the 16S V6 hypervariable region. The primers for the first PCR incorporate the barcode sequences and the sequencing adapters, while the primers for the second PCR adds the Illumina flow cell adapters. The amplicons are purified/quantified and equal quantities from each sample are pooled, gel extracted and sequenced. (B) Schematic detailing the regions that are sequenced during the 100 bp paired-end sequencing reaction.

Supplementary Table 1: Characteristics of IBD patients and control subjects employed in the current study.

Sample ID	Gender	Age	Description	Disease	Disease Location	Paris Classification	Visual Appearance	PCDAI /PUCAI	Experiment
HMC002CO	F	16	UC	IBD	UlcerativeProctitis	E1S0	Normal	mild(20)	Illumina
HMC003CO	F	9	Control	Control	NA	NA	Normal	NA	Illumina
HMC004CO	M	12	Control	Control	NA	NA	Normal	NA	454Pyro
HMC005CO	F	10	Control	Control	NA	NA	Normal	NA	Illumina
HMC006CO	M	15	Control	Control	NA	NA	Normal	NA	Illumina
HMC012CO	M	13	CD	IBD	Ileal	A1bL1B1G0	Normal	mild(7.5)	Illumina
HMC013CO	M	12	UC	IBD	Pancolitis	E4S1	Inflammed	Severe(65)	Illumina
HMC014CO	F	14	CD	IBD	Ileal_Colonic	A1bL3L4aB1G1	Normal	Moderate(37.5)	Illumina/454pyro/qPCR
HMC015CO	F	14	CD	IBD	Ileal	A1bL1B2G0	Normal	mild(10)	Illumina
HMC016CO	M	13	CD	IBD	Ileal_Colonic	A1bL3B1G0	Normal	mild(20)	Illumina/qPCR
HMC017CO	M	13	CD	IBD	Colonic	A1bL2L4aB1G1	Inflammed	Severe(57.5)	Illumina/qPCR
HMC018CO	M	13	Control	Control	NA	NA	Normal	NA	Illumina/qPCR
HMC019CO	M	14	UC	IBD	Pancolitis	E4S1	Inflammed	Severe(80)	Illumina/454pyro
HMC020CO	F	12	Control	Control	NA	NA	Normal	NA	454Pyro
HMC022CO	M	14	CD	IBD	Ileal	A1bL1B1G1	Normal	Moderate(37.5)	Illumina/qPCR
HMC023CO	M	14	UC	IBD	ExtensiveUC	E3S1	Normal	Moderate(50)	Illumina/454pyro
HMC024CO	M	16	UC	IBD	ExtensiveUC	E3S1	Normal	Moderate(40)	Illumina/454pyro/Ion Torrent
HMC025CO	F	15	CD	IBD	Upper_GIT	A1bL4bB1G0	Normal	mild(20)	Illumina
HMC026CO	F	16	Control	Control	NA	NA	Normal	NA	454Pyro
HMC027CO	F	16	Control	Control	NA	NA	Normal	NA	Illumina
HMC028CO	M	13	Control	Control	NA	NA	Normal	NA	Illumina/454pyro/qPCR/Ion Torrent
HMC029CO	M	14	CD	IBD	Ileal_Colonic	A1bL3L4aB1G0	Inflammed	Moderate(35)	Illumina/qPCR
HMC030CO	F	17	CD	IBD	Ileal_Colonic	A1bL4aL3B1G0	Inflammed	Severe(45)	Illumina
HMC038CO	F	16	CD	IBD	Colonic	A1bL2B1pG0	Inflammed	mild(20)	Illumina/qPCR
HMC039CO	F	13	CD	IBD	Ileal	A1bL1L4aB1G1	Normal	Severe(60)	Illumina/qPCR
HMC041CO	F	15	CD	IBD	Ileal_Colonic	A1bL3B1G0	Normal	Severe(62.5)	Illumina/qPCR
HMC042CO	M	17	Control	Control	NA	NA	Normal	NA	Illumina
HMC043CO	F	16	Control	Control	NA	NA	Normal	NA	Illumina/qPCR
HMC044CO	F	15	CD	IBD	Ileal	A1bL1B3G1	Normal	Severe(52.5)	Illumina

Sample ID	Gender	Age	Description	Disease	Disease Location	Paris Classification	Visual Appearance	PCDAI /PUCAI	Experiment
HMC045CO	M	17	UC	IBD	Pancolitis	E4S0	Inflammed	Moderate(45)	Illumina/qPCR
HMC046CO	F	4	UC	IBD	Pancolitis	E4S1	Inflammed	Severe(65)	Illumina/454pyro
HMC047CO	M	16	CD	IBD	Ileal_Colonic	A1bL3L4aB1G1	Inflammed	Severe(65)	Illumina/qPCR
HMC049CO	M	12	CD	IBD	Ileal	A1bL1B1G1	Normal	Severe(40)	Illumina/454pyro/qPCR
HMC050CO	F	16	CD	IBD	Ileal	A1bL1B1G0	Normal	mild(12.5)	Illumina
HMC051CO	F	16	CD	IBD	Ileal_Colonic	A1bL3L4aB1G0	Inflammed	Severe(65)	Illumina/qPCR
HMC052CO	F	8	Control	Control	NA	NA	Normal	NA	454Pyro
HMC055CO	M	6	Control	Control	NA	NA	Normal	NA	Illumina/454pyro/qPCR
HMC056CO	F	8	Control	Control	NA	NA	Normal	NA	Illumina
HMC059CO	M	14	Control	Control	NA	NA	Normal	NA	Illumina
HMC061CO	M	9	CD	IBD	Colonic	A1aL2B1G0	Inflammed	Moderate(32.5)	Illumina/454pyro/qPCR
HMC062CO	F	15	CD	IBD	Ileal_Colonic	A1bL3L4aB3G0	Inflammed	Severe(57.5)	Illumina/qPCR
HMC063CO	M	13	CD	IBD	Ileal	A1bL1L4aB1G1	Normal	Severe(65)	Illumina/454pyro/qPCR
HMC064CO	M	18	UC	IBD	Pancolitis	E4S1	Inflammed	mild(0)	Illumina/Ion Torrent
HMC065CO	M	16	CD	IBD	Ileal	A1bL1B2B3G0	Normal	Severe(50)	Illumina
HMC066CO	F	18	UC	IBD	Pancolitis	E4S0	Inflammed	Moderate(35)	Illumina/454pyro
HMC068CO	M	17	CD	IBD	Colonic	A1bL2B1G0	Inflammed	Moderate(32.5)	454Pyro
HMC069CO	F	17	Control	Control	NA	NA	Normal	NA	Illumina
HMC070CO	F	8	Control	Control	NA	NA	Normal	NA	454Pyro
HMC071CO	M	11	Control	Control	NA	NA	Normal	NA	Illumina
HMC072CO	M	12	CD	IBD	Ileal_Colonic	A1bL3L4aB1G1	Inflammed	Severe(55)	Illumina/qPCR
HMC073CO	F	16	Control	Control	NA	NA	Normal	NA	Illumina
HMC074CO	F	16	Control	Control	NA	NA	Normal	NA	454Pyro
HMC075CO	M	14	CD	IBD	Colonic	A1bL2B1G1	Inflammed	Severe(55)	Illumina
HMC076CO	F	17	UC	IBD	Pancolitis	E4S0	Inflammed	Moderate(55)	454pyro
HMC077CO	M	17	UC	IBD	ExtensiveUC	E3S0	Normal	Moderate(50)	Illumina/454pyro
HMC078CO	F	15	CD	IBD	Ileal	A1bL1B1G0	Normal	Severe(45)	Illumina/qPCR
HMC079CO	M	16	CD	IBD	Ileal_Colonic	A1bL3B1pG0	Inflammed	Severe(50)	Illumina/qPCR
HMC081CO	M	11	CD	IBD	Ileal_Colonic	A1bL3L4aB1G1	Normal	Severe(67.5)	Illumina
HMC082CO	F	15	CD	IBD	Ileal_Colonic	A1aL3B1G0	Inflammed	mild(27.5)	Illumina
HMC084CO	M	16	CD	IBD	Colonic	A1bL2B1G0	Inflammed	Severe(42.5)	Illumina

Sample ID	Gender	Age	Description	Disease	Disease Location	Paris Classification	Visual Appearance	PCDAI /PUCAI	Experiment
HMC085CO	M	16	CD	IBD	Colonic	A1bL2B1pG1	Normal	Severe(67.5)	Illumina/454pyro/qPCR
HMC087CO	F	18	Control	Control	NA	NA	Normal	NA	Illumina/454pyro/qPCR/Ion Torrent
HMC088CO	M	16	UC	IBD	UlcerativeProctitis	E1S0	Normal	mild(20)	Illumina
HMC090CO	M	16	CD	IBD	Ileal_Colonic	A1bL3L4aB1G0	Inflammed	Severe(52.5)	Illumina/454pyro/qPCR
HMC091CO	M	12	Control	Control	NA	NA	Normal	NA	Illumina
HMC092CO	M	12	UC	IBD	ExtensiveUC	E3S1	Normal	Severe(75)	454pyro
HMC093CO	M	12	CD	IBD	Ileal	A1bL1B1pG0	Normal	mild(27.5)	Illumina/454pyro
HMC095CO	M	11	CD	IBD	Ileal_Colonic	A1bL3B1pG1	Normal	Severe(65)	Illumina
HMC097CO	M	12	CD	IBD	Ileal_Colonic	A1bL3L4aB1G0	Inflammed	Severe(40)	454Pyro
HMC098CO	F	16	Control	Control	NA	NA	Normal	NA	Illumina/qPCR
HMC102CO	F	17	Control	Control	NA	NA	Normal	NA	Illumina/Ion Torrent
HMC106CO	F	16	Control	Control	NA	NA	Normal	NA	qRTPCR/qPCR
HMC109CO	F	3	Control	Control	NA	NA	Normal	NA	qRTPCR/qPCR
HMC110CO	F	4	Control	Control	NA	NA	Normal	NA	qPCR
HMC112CO	M	9	Control	Control	NA	NA	Normal	NA	qRTPCR/qPCR
HMC113DC	M	15	UC	IBD	Pancolitis	E4S1	Inflammed	Inactive(0)	qRTPCR
HMC117CO	F	15	Control	Control	NA	NA	Normal	NA	qRTPCR/qPCR
HMC201CO	F	11	CD	IBD	Colonic	A1bL2B1G1	Inflammed	Severe(47.5)	Illumina/Massspec/qRTPCR/qPCR/Ion Torrent
HMC202CO	M	17	CD	IBD	Ileal_Colonic	A1bL3B1G0	Inflammed	Moderate(37.5)	Illumina/qRTPCR/Ion Torrent
HMC203CO*	M	11	CD	IBD	Ileal_Colonic	A1bL3B1G0	Inflammed	Severe(55)	Illumina/qRTPCR/qPCR/Ion Torrent
HMC204CO	M	13	CD	IBD	Ileal	A1bL1L4bB1G1	Normal	Severe(50)	Illumina/qRTPCR
HMC205CO	M	13	CD	IBD	Ileal	A1bL1L4aB1pG1	Normal	Severe(45)	Illumina/qRTPCR/qPCR
HMC206CO	M	14	CD	IBD	Ileal_Colonic	A1bL3B1G1	Inflammed	Severe(47.5)	Illumina/qPCR/qRTPCR
HMC207CO	F	13	UC	IBD	Pancolitis	E4S1	Inflammed	Severe(70)	Illumina/Ion Torrent
HMC208CO	F	16	Control	Control	NA	NA	Normal	NA	Illumina/qPCR/qRTPCR/Ion Torrent
HMC210CO	F	17	CD	IBD	Ileal	A1bL1B1G0	Normal	Moderate(32.5)	qRTPCR
HMC211CO	M	13	Control	Control	NA	NA	Normal	NA	qRTPCR
HMC212CO	F	15	Control	Control	NA	NA	Normal	NA	qRTPCR/qPCR
HMC213CO	M	10	CD	IBD	Ileal_Colonic	A1bL3B1G1	Inflammed	Severe(57.5)	Illumina/MassSpec
HMC214CO	M	17	UC	IBD	Pancolitis	E4S0	Inflammed	Moderate(40)	qRTPCR
HMC215CO	F	12	UC	IBD	Pancolitis	E4S1	Inflammed	Severe(65)	qRTPCR

Sample ID	Gender	Age	Description	Disease	Disease Location	Paris Classification	Visual Appearance	PCDAI /PUCAI	Experiment
HMC217CO	F	14	CD	IBD	Ileal	A1bL1L4bB1G0	Normal	Severe(52.5)	qRTPCR/qPCR
HMC219CO	M	14	CD	IBD	Ileal_Colonic	A1bL3B1G1	Inflammed	Moderate(37.5)	qRTPCR/qPCR
HMC220CO	M	13	CD	IBD	Ileal_Colonic	A1bL3B1G0	Inflammed	Severe(65)	Illumina/qRTPCR/Ion Torrent
HMC221CO	F	9	Control	Control	NA	NA	Normal	NA	Illumina/qPCR/qRTPCR
HMC222CO	M	10	Control	Control	NA	NA	Normal	NA	Illumina/qPCR/qRTPCR
HMC223CO	F	9	CD	IBD	Colonic	A1aL2B1G1	Inflammed	Severe(50)	Illumina/MassSpec/qRTPCR/qPCR/Ion Torrent
HMC225CO	F	15	Control	Control	NA	NA	Normal	NA	Illumina/qPCR/qRTPCR
HMC228CO	M	13	CD	IBD	Ileal_Colonic	A1bL3B1pG0	Inflammed	Severe(62.5)	Illumina/qRTPCR/Ion Torrent
HMC229CO	M	10	CD	IBD	Colonic	A1bL2L4abB1G0	Inflammed	Severe(57.5)	Illumina/qRTPCR/MassSpec/qPCR
HMC230CO	M	14	CD	IBD	Ileal_Colonic	A1bL3L4aB1pG0	Inflammed	Severe(52.5)	Illumina/qRTPCR/MassSpec/Ion Torrent
HMC231CO	F	15	CD	IBD	Ileal	A1bL1B1G0	Normal	Moderate(37.5)	Illumina/qPCR/qRTPCR
HMC232CO	F	15	Control	Control	NA	NA	Normal	NA	Illumina/qPCR/qRTPCR/Ion Torrent
HMC234CO	F	13	CD	IBD	Ileal_Colonic	A1bL3B1G0	Inflammed	Severe(52.5)	qRTPCR
HMC235CO	M	11	CD	IBD	Ileal	A1bL1B1G0	Normal	Moderate(32.5)	Illumina/qRTPCR
HMC237CO	M	14	Control	Control	NA	NA	Normal	NA	Illumina/qRTPCR/qPCR
HMC238CO	M	14	Control	Control	NA	NA	Normal	NA	Illumina/qPCR/qRTPCR/MassSpec/Ion Torrent
HMC240CO	M	10	CD	IBD	Ileal	A1bL1B1G0	Normal	Severe(70)	Illumina/qRTPCR
HMC241CO	F	12	Control	Control	NA	NA	Normal	NA	qRTPCR
HMC242CO	F	13	UC	IBD	Pancolitis	E4S1	Inflammed	Severe(85)	Illumina/Ion Torrent
HMC243CO	M	8	Control	Control	NA	NA	Normal	NA	Illumina/qRTPCR/MassSpec/Ion Torrent
HMC244CO	M	9	UC	IBD	Pancolitis	E4S1	Inflammed	Severe(75)	Illumina/qRTPCR/Ion Torrent
HMC245CO	M	16	Control	Control	NA	NA	Normal	NA	Illumina/qRTPCR/qPCR/Ion Torrent
HMC246CO	M	13	Control	Control	NA	NA	Normal	NA	Illumina/qRTPCR/qPCR/Ion Torrent
HMC247CO	F	13	UC	IBD	Pancolitis	E4S1	Inflammed	Severe(80)	Illumina/qRTPCR/Ion Torrent
HMC249CO	M	14	UC	IBD	Pancolitis	E4S0	Inflammed	Moderate(45)	Illumina/qRTPCR/Ion Torrent
HMC251CO	M	7	CD	IBD	Ileal_Colonic	A1aL3L4aB1G0	Inflammed	Severe(47.5)	Illumina/MassSpec/Ion Torrent
HMC252CO	M	9	CD	IBD	Ileal_Colonic	A1aL3B1G0	Inflammed	Moderate(37.5)	MassSpec
HMC253CO	F	11	CD	IBD	Colonic	A1bL4aL2B1G0	Inflammed	Severe(40)	Illumina/qPCR/Ion Torrent
HMC255CO	M	13	Control	Control	NA	NA	Normal	NA	Illumina/qPCR/Ion Torrent
HMC256CO	F	10	CD	IBD	Colonic	A1bL2B1G0	Normal	Moderate(30)	Illumina

Sample ID	Gender	Age	Description	Disease	Disease Location	Paris Classification	Visual Appearance	PCDAI /PUCAI	Experiment
HMC258CO	M	15	Control	Control	NA	NA	Normal	NA	Illumina/qPCR/MassSpec/Ion Torrent
HMC260CO	M	12	CD	IBD	Colonic	A1bL2B1pG0	Inflammed	Severe(47.5)	Illumina/qPCR/Ion Torrent
HMC261CO	F	13	CD	IBD	Ileal_Colonic	A1bL3L4abB1pG0	Normal	Moderate(37.5)	Illumina/qPCR
HMC262CO	M	15	CD	IBD	Ileal	A1bL1L4aB1pG0	Normal	mild(27.5)	Illumina
HMC265CO	M	14	Control	Control	NA	NA	Normal	NA	Illumina/MassSpec/qPCR/Ion Torrent
HMC270CO	F	17	Control	Control	NA	NA	Normal	NA	Illumina/qPCR/qPCR/Ion Torrent
HMC272CO	M	12	CD	IBD	Ileal_Colonic	A1bL3B1PG1	Inflammed	Severe(65)	Illumina
HMC273CO	M	15	UC	IBD	LeftSidedUC	E2S0	Normal	Moderate(40)	Illumina/qPCR/Ion Torrent
HMC274CO	M	17	UC	IBD	Pancolitis	E4S1	Inflammed	Severe(70)	Illumina/qPCR/Ion Torrent
HMC277CO	F	17	CD	IBD	Ileal_Colonic	A1bL3L4abB1G0	Inflammed	Moderate(32.5)	MassSpec
HMC279CO	F	14	CD	IBD	Colonic	A1bL2B1G0	Inflammed	Severe(77.5)	Illumina/Ion Torrent
HMC280CO	M	15	CD	IBD	Ileal_Colonic	A1bL3B1G0	Inflammed	Moderate(45)	Illumina/MassSpec
HMC281CO	F	15	Control	Control	NA	NA	Normal	NA	Illumina/qPCR/MassSpec
HMC283CO	M	13	Control	Control	NA	NA	Normal	NA	qPCR
HMC288CO	M	14	CD	IBD	Ileal_Colonic	A1bL3B1pG0	Inflammed	Severe(67.5)	Illumina/qPCR/Ion Torrent
HMC289CO	M	12	CD	IBD	Ileal_Colonic	A1bL3B1pG0	Inflammed	Severe(67.5)	Illumina/qPCR
HMC295CO	F	14	CD	IBD	Colonic	A1bL2B1G0	Inflammed	mild(25)	Illumina/qPCR
HMC296CO	M	16	Control	Control	NA	NA	Normal	NA	Illumina/qPCR
HMC297CO	F	17	Control	Control	NA	NA	Normal	NA	Illumina/qPCR
HMC298CO*	M	12	CD	IBD	Ileal	A1bL1B1G0	Normal	Severe(62.5)	Illumina/qPCR
HMC299CO	M	11	UC	IBD	Pancolitis	E4S0	Inflammed	Moderate(40)	Illumina/qPCR
HMC301CO	F	14	CD	IBD	Ileal_Colonic	A1bL3L4aB1G0	Inflammed	Severe(60)	Illumina/qPCR/MassSpec
HMC302CO	F	17	Control	Control	NA	NA	Normal	NA	Illumina/qPCR/MassSpec
HMC303CO	F	15	UC	IBD	Pancolitis	E4S0	Inflammed	Moderate(45)	Illumina/qPCR/Ion Torrent
HMC305CO	M	4	CD	IBD	Ileal_Colonic	A1aL3B1pG0	Normal	mild(12.5)	qPCR
HMC307CO	F	16	Control	Control	NA	NA	Normal	NA	Illumina/qPCR/MassSpec
HMC308CO	M	10	Control	Control	NA	NA	Normal	NA	qPCR
HMC309CO	M	10	Control	Control	NA	NA	Normal	NA	Illumina/qPCR
HMC310CO	F	9	UC	IBD	Pancolitis	E4S0	Inflammed	Moderate(35)	Illumina
HMC311CO	F	15	CD	IBD	Ileal	A1bL1B1pG0	Normal	Moderate(32.5)	Illumina/qPCR/MassSpec
HMC313CO	M	16	Control	Control	NA	NA	Normal	NA	Illumina/qPCR/MassSpec

Sample ID	Gender	Age	Description	Disease	Disease Location	Paris Classification	Visual Appearance	PCDAI /PUCAI	Experiment
HMC315CO	M	16	Control	Control	NA	NA	Normal	NA	Illumina/qPCR/MassSpec
HMC316CO	M	14	CD	IBD	Ileal_Colonic	A1bL3L4aB1G0	Inflammed	mild(27.5)	Illumina/qPCR/MassSpec
HMC319CO	M	12	CD	IBD	Ileal	A1bL1B1G1	Normal	Severe(45)	Illumina/qPCR
HMC321CO	F	16	Control	Control	NA	NA	Normal	NA	Illumina/qPCR/MassSpec
HMC323CO	F	16	CD	IBD	Ileal_Colonic	A1bL3B1G0	Inflammed	Severe(55)	Illumina/MassSpec
HMC345CO	M	17	CD	IBD	Ileal_Colonic	A1bL3L4aB1G0	Inflammed	Severe(57.5)	qPCR
HMC348CO	F	17	UC	IBD	ExtensiveUC	E3S1	Normal	Severe(85)	qPCR
HMC350CO	M	13	Control	Control	NA	NA	Normal	NA	qPCR
HMC351CO	M	17	UC	IBD	Pancolitis	E4S0	Inflammed	Moderate(60)	qPCR
HMC354CO	F	15	UC	IBD	Pancolitis	E4S0	Inflammed	mild(25)	qPCR
HMC359CO	M	11	CD	IBD	Ileal	A1bL1B1pG0	Normal	Moderate(37.5)	qPCR
HMC360CO	F	15	UC	IBD	Pancolitis	E4S1	Inflammed	Severe(65)	qPCR
HMC361CO	F	14	UC	IBD	Pancolitis	E4S1	Inflammed	Severe(75)	qPCR
HMC363CO	M	14	CD	IBD	Ileal	A1bL1B1pG0	Normal	mild(20)	qPCR
HMC369CO	F	17	UC	IBD	Pancolitis	E4S1	Inflammed	Severe(75)	qPCR
HMC371CO	M	7	Control	Control	NA	NA	Normal	NA	qPCR
HMC372CO	M	14	Control	Control	NA	NA	Normal	NA	qPCR
HMC374CO	M	15	CD	IBD	Ileal_Colonic	A1bL3B2G0	Normal	Moderate(37.5)	qPCR
HMC377CO	F	10	CD	IBD	Colonic	A1bL2L4aB1G0	Inflammed	Moderate(37.5)	qPCR
HMC378CO	F	8	CD	IBD	Ileal_Colonic	A1aL3L4aB1G1	Inflammed	Moderate(30)	qPCR/MassSpec
HMC379CO	F	10	CD	IBD	Ileal	A1bL1B1pG0	Normal	mild(27.5)	qPCR/MassSpec
HMC381CO	M	17	Control	Control	NA	NA	Normal	NA	qPCR
HMC382CO	F	13	UC	IBD	Pancolitis	E4S1	Inflammed	Severe(65)	qPCR
HMC390CO	F	8	CD	IBD	Ileal-Colonic	A1aL3L4aB2G1	Inflammed	Moderate	qPCR
HMC391CO	M	15	CD	IBD	Ileal	A1bL1BIpG1	Normal	Inactive(5)	qPCR
HMC392CO	M	15	CD	IBD	Ileal_Colonic	A1bL3B1pG0	Normal	Inactive(0)	qPCR
HMC394CO	M	16	CD	IBD	Ileal_Colonic	A1bL3B1G0	Inflammed	Moderate(32.5)	qPCR
HMC396CO	M	17	CD	IBD	Ileal_Colonic	A1bL3B1G1	Inflammed	Inactive(2.5)	qPCR
HMC397CO	F	10	Control	Control	NA	NA	Normal	NA	qPCR
HMC398CO	F	12	UC	IBD	Pancolitis	E4S1	Inflammed	Severe(70)	qPCR
HMC399CO	F	14	CD	IBD	Colonic	A1bL2B1G0	Inflammed	mild(17.5)	qPCR

Sample ID	Gender	Age	Description	Disease	Disease Location	Paris Classification	Visual Appearance	PCDAI /PUCAI	Experiment
HMC400CO	M	13	UC	IBD	LeftSidedUC	E2S0	Normal	Moderate(60)	qPCR
HMC401CO	M	12	CD	IBD	Ileal	A1bL1B2G0	Normal	mild(10)	qPCR
HMC402CO	F	7	UC	IBD	Pancolitis	E4S0	Inflammed	Moderate(60)	qPCR
HMC403CO	F	16	Control	Control	NA	NA	Normal	NA	qPCR
HMC405CO	M	15	UC	IBD	Pancolitis	E4S0	Inflammed	Moderate(55)	qPCR
HMC406CO	F	4	CD	IBD	Colonic	A1aL2L4aB1G0	Inflammed	Moderate(37.5)	MassSpec
HMC407CO	M	15	CD	IBD	Ileal_Colonic	A1bL3L4aB1pGO	Normal	Moderate(32.5)	MassSpec
HMC418CO	M	10	CD	IBD	Ileal	A1aL1L4aB1G0	Normal	mild(12.5)	MassSpec
HMC449CO	M	16	CD	IBD	Ileal_Colonic	A1bL3B1G1	Inflammed	mild(17.5)	MassSpec
HMC460CO	M	17	CD	IBD	Ileal_Colonic	A1bL3B1G0	Inflammed	mild(22.5)	MassSpec
HMC465CO	M	11	CD	IBD	Ileal_Colonic	A1bL4aL3B1G1	Inflammed	Moderate(35)	MassSpec

CD = Crohn's disease; M= Male; F= Female; UC = Ulcerative Colitis; Illumina = Hiseq2500 sequencing; 454pyro = pyrosequencing Roche 454 GS-FLX; PUCAI = Pediatric Ulcerative Colitis Activity Index and PCDAI = Pediatric Crohn’s Disease Activity Index.

*Patient HMC203 received three days of metronidazole treatment prior to colonoscopy and patient HM298 received four days of mesalazine treatment followed by six days of no treatment prior to colonoscopy

Supplementary Table 2: Primers used for constructing the 16S rRNA-V6 Illumina library. Nucleotides in bold represent the barcode sequences.

Primer Name	Primer Sequence (5` – 3`)
**MF1**	ACACTCTTTCCCTACACGACGCTCTTCCGATCT**ATAGCG**AAACTCAAAKGAATTGACGG
**MF2**	ACACTCTTTCCCTACACGACGCTCTTCCGATCT**AGGGT**AAACTCAAAKGAATTGACGG
**MF3**	ACACTCTTTCCCTACACGACGCTCTTCCGATCT**TTCAT**AAACTCAAAKGAATTGACGG
**MF4**	ACACTCTTTCCCTACACGACGCTCTTCCGATCT**GATCGT**AAACTCAAAKGAATTGACGG
**MF5**	ACACTCTTTCCCTACACGACGCTCTTCCGATCT**GCCCGT**AAACTCAAAKGAATTGACGG
**MF6**	ACACTCTTTCCCTACACGACGCTCTTCCGATCT**CTGTC**AAACTCAAAKGAATTGACGG
**MF7**	ACACTCTTTCCCTACACGACGCTCTTCCGATCT**CACGT**AAACTCAAAKGAATTGACGG
**MF8**	ACACTCTTTCCCTACACGACGCTCTTCCGATCT**CGTACG**AAACTCAAAKGAATTGACGG
**MF9**	ACACTCTTTCCCTACACGACGCTCTTCCGATCT**GGAC**AAACTCAAAKGAATTGACGG
**MF10**	ACACTCTTTCCCTACACGACGCTCTTCCGATCT**TAGA**AAACTCAAAKGAATTGACGG
**MF11**	ACACTCTTTCCCTACACGACGCTCTTCCGATCT**TCAT**AAACTCAAAKGAATTGACGG
**MF12**	ACACTCTTTCCCTACACGACGCTCTTCCGATCT**ACTT**AAACTCAAAKGAATTGACGG
**MR1**	CTCGGCATTCCTGCTGAACCGCTCTTCCGATCT**ATAGCGA**ACGAGCTGACGACARCCATG
**MR2**	CTCGGCATTCCTGCTGAACCGCTCTTCCGATCT**AGGGTA**ACGAGCTGACGACARCCATG
**MR3**	CTCGGCATTCCTGCTGAACCGCTCTTCCGATCT**TTCATA**ACGAGCTGACGACARCCATG
**MR4**	CTCGGCATTCCTGCTGAACCGCTCTTCCGATCT**GATCGTA**ACGAGCTGACGACARCCATG
**MR5**	CTCGGCATTCCTGCTGAACCGCTCTTCCGATCT**GCCCGTA**ACGAGCTGACGACARCCATG
**MR6**	CTCGGCATTCCTGCTGAACCGCTCTTCCGATCT**CTGTCA**ACGAGCTGACGACARCCATG
**MR7**	CTCGGCATTCCTGCTGAACCGCTCTTCCGATCT**CACGTA**ACGAGCTGACGACARCCATG
**MR8**	CTCGGCATTCCTGCTGAACCGCTCTTCCGATCT**CGTACGA**ACGAGCTGACGACARCCATG
**MR9**	CTCGGCATTCCTGCTGAACCGCTCTTCCGATCT**GGACA**ACGAGCTGACGACARCCATG
**MR10**	CTCGGCATTCCTGCTGAACCGCTCTTCCGATCT**TAGAA**ACGAGCTGACGACARCCATG
**MR11**	CTCGGCATTCCTGCTGAACCGCTCTTCCGATCT**TCAT**ACGAGCTGACGACARCCATG
**MR12**	CTCGGCATTCCTGCTGAACCGCTCTTCCGATCT**ACTT**ACGAGCTGACGACARCCATG
**PCR-FWD1**	AATGATACGGCGACCACCGAGATCTACACTCTTTCCCTACACGACGCTCTTCCGATCT
**PCR-RVS1**	CAAGCAGAAGACGCCATACGAGATCGGTCTCGGCATTCCTGCTGAACCGCTCTTCCGATCT

Supplementary Table 3: Primers used for constructing the 16S rRNA-V4/V6 Ion Torrent libraries

Primer Name	16S Target Region	Barcode	Primer Sequence (5' – 3')
v6_BC001	V6	TCTTAACCGGC	CCATCTCATCCCTGCGTGTCTCCGACTCAGTCTTAACCGGCAAACTCAAAKGAATTGACGG
v4_BC001	V4	TCTTAACCGGC	CCATCTCATCCCTGCGTGTCTCCGACTCAGTCTTAACCGGCAYTGGGYDTAAAGNG
v6_BC002	V6	TTAGGTACGGC	CCATCTCATCCCTGCGTGTCTCCGACTCAGTTAGGTACGGCAAACTCAAAKGAATTGACGG
v4_BC002	V4	TTAGGTACGGC	CCATCTCATCCCTGCGTGTCTCCGACTCAGTTAGGTACGGCAYTGGGYDTAAAGNG
v6_BC003	V6	TTCGTAACCGC	CCATCTCATCCCTGCGTGTCTCCGACTCAGTTCGTAACCGCAAACTCAAAKGAATTGACGG
v4_BC003	V4	TTCGTAACCGC	CCATCTCATCCCTGCGTGTCTCCGACTCAGTTCGTAACCGCAYTGGGYDTAAAGNG
v6_BC004	V6	TTCCGTAAGCC	CCATCTCATCCCTGCGTGTCTCCGACTCAGTTCCGTAAGCCAAACTCAAAKGAATTGACGG
v4_BC004	V4	TTCCGTAAGCC	CCATCTCATCCCTGCGTGTCTCCGACTCAGTTCCGTAAGCCAYTGGGYDTAAAGNG
v6_BC005	V6	TTACGGACGAC	CCATCTCATCCCTGCGTGTCTCCGACTCAGTTACGGACGACAAACTCAAAKGAATTGACGG
v4_BC005	V4	TTACGGACGAC	CCATCTCATCCCTGCGTGTCTCCGACTCAGTTACGGACGACAYTGGGYDTAAAGNG
v6_BC006	V6	TACTAACCGCC	CCATCTCATCCCTGCGTGTCTCCGACTCAGTACTAACCGCCAAACTCAAAKGAATTGACGG
v4_BC006	V4	TACTAACCGCC	CCATCTCATCCCTGCGTGTCTCCGACTCAGTACTAACCGCCAYTGGGYDTAAAGNG
v6_BC008	V6	TTAACCGCACC	CCATCTCATCCCTGCGTGTCTCCGACTCAGTTAACCGCACCAAACTCAAAKGAATTGACGG
v4_BC008	V4	TTAACCGCACC	CCATCTCATCCCTGCGTGTCTCCGACTCAGTTAACCGCACCAYTGGGYDTAAAGNG
v6_BC009	V6	TTAACGACGCC	CCATCTCATCCCTGCGTGTCTCCGACTCAGTTAACGACGCCAAACTCAAAKGAATTGACGG
v4_BC009	V4	TTAACGACGCC	CCATCTCATCCCTGCGTGTCTCCGACTCAGTTAACGACGCCAYTGGGYDTAAAGNG
v6_BC010	V6	TTAACCGTGGC	CCATCTCATCCCTGCGTGTCTCCGACTCAGTTAACCGTGGCAAACTCAAAKGAATTGACGG
v4_BC010	V4	TTAACCGTGGC	CCATCTCATCCCTGCGTGTCTCCGACTCAGTTAACCGTGGCAYTGGGYDTAAAGNG
v6_BC011	V6	TATTACCGGCC	CCATCTCATCCCTGCGTGTCTCCGACTCAGTATTACCGGCCAAACTCAAAKGAATTGACGG
v4_BC011	V4	TATTACCGGCC	CCATCTCATCCCTGCGTGTCTCCGACTCAGTATTACCGGCCAYTGGGYDTAAAGNG
v6_BC012	V6	TAACGGCCTTC	CCATCTCATCCCTGCGTGTCTCCGACTCAGTAACGGCCTTCAAACTCAAAKGAATTGACGG
v4_BC012	V4	TAACGGCCTTC	CCATCTCATCCCTGCGTGTCTCCGACTCAGTAACGGCCTTCAYTGGGYDTAAAGNG
v6_BC013	V6	TCCTAACGTCC	CCATCTCATCCCTGCGTGTCTCCGACTCAGTCCTAACGTCCAAACTCAAAKGAATTGACGG
v4_BC013	V4	TCCTAACGTCC	CCATCTCATCCCTGCGTGTCTCCGACTCAGTCCTAACGTCCAYTGGGYDTAAAGNG
v6_BC014	V6	AATACCGGACC	CCATCTCATCCCTGCGTGTCTCCGACTCAGAATACCGGACCAAACTCAAAKGAATTGACGG
v4_BC014	V4	AATACCGGACC	CCATCTCATCCCTGCGTGTCTCCGACTCAGAATACCGGACCAYTGGGYDTAAAGNG
v6_BC015	V6	TTACCTTCCGC	CCATCTCATCCCTGCGTGTCTCCGACTCAGTTACCTTCCGCAAACTCAAAKGAATTGACGG
v4_BC015	V4	TTACCTTCCGC	CCATCTCATCCCTGCGTGTCTCCGACTCAGTTACCTTCCGCAYTGGGYDTAAAGNG
v6_BC016	V6	TACTACGGTCC	CCATCTCATCCCTGCGTGTCTCCGACTCAGTACTACGGTCCAAACTCAAAKGAATTGACGG
v4_BC016	V4	TACTACGGTCC	CCATCTCATCCCTGCGTGTCTCCGACTCAGTACTACGGTCCAYTGGGYDTAAAGNG
v6_BC017	V6	TAACCGACTCC	CCATCTCATCCCTGCGTGTCTCCGACTCAGTAACCGACTCCAAACTCAAAKGAATTGACGG
v4_BC017	V4	TAACCGACTCC	CCATCTCATCCCTGCGTGTCTCCGACTCAGTAACCGACTCCAYTGGGYDTAAAGNG
v6_BC018	V6	TTCCGGTATCC	CCATCTCATCCCTGCGTGTCTCCGACTCAGTTCCGGTATCCAAACTCAAAKGAATTGACGG
v4_BC018	V4	TTCCGGTATCC	CCATCTCATCCCTGCGTGTCTCCGACTCAGTTCCGGTATCCAYTGGGYDTAAAGNG
v6_BC019	V6	TTAACACCGGC	CCATCTCATCCCTGCGTGTCTCCGACTCAGTTAACACCGGCAAACTCAAAKGAATTGACGG
v4_BC019	V4	TTAACACCGGC	CCATCTCATCCCTGCGTGTCTCCGACTCAGTTAACACCGGCAYTGGGYDTAAAGNG
v6_BC020	V6	TTAATCCGGCC	CCATCTCATCCCTGCGTGTCTCCGACTCAGTTAATCCGGCCAAACTCAAAKGAATTGACGG
v4_BC020	V4	TTAATCCGGCC	CCATCTCATCCCTGCGTGTCTCCGACTCAGTTAATCCGGCCAYTGGGYDTAAAGNG

Primer Name	16S Target Region	Barcode	Primer Sequence (5' – 3')
v6_BC023	V6	AACCTCGGTTG	CCATCTCATCCCTGCGTGTCTCCGACTCAGAACCTCGGTTGAAACTCAAAKGAATTGACGG
v4_BC023	V4	AACCTCGGTTG	CCATCTCATCCCTGCGTGTCTCCGACTCAGAACCTCGGTTGAYTGGGYDTAAAGNG
v6_BC024	V6	TCTCCGTTAGG	CCATCTCATCCCTGCGTGTCTCCGACTCAGTCTCCGTTAGGAAACTCAAAKGAATTGACGG
v4_BC024	V4	TCTCCGTTAGG	CCATCTCATCCCTGCGTGTCTCCGACTCAGTCTCCGTTAGGAYTGGGYDTAAAGNG
v6_BC025	V6	TTATTCCGCCG	CCATCTCATCCCTGCGTGTCTCCGACTCAGTTATTCCGCCGAAACTCAAAKGAATTGACGG
v4_BC025	V4	TTATTCCGCCG	CCATCTCATCCCTGCGTGTCTCCGACTCAGTTATTCCGCCGAYTGGGYDTAAAGNG
v6_BC026	V6	TTAAGGTGCCG	CCATCTCATCCCTGCGTGTCTCCGACTCAGTTAAGGTGCCGAAACTCAAAKGAATTGACGG
v4_BC026	V4	TTAAGGTGCCG	CCATCTCATCCCTGCGTGTCTCCGACTCAGTTAAGGTGCCGAYTGGGYDTAAAGNG
v6_BC027	V6	TAATTGGCCGG	CCATCTCATCCCTGCGTGTCTCCGACTCAGTAATTGGCCGGAAACTCAAAKGAATTGACGG
v4_BC027	V4	TAATTGGCCGG	CCATCTCATCCCTGCGTGTCTCCGACTCAGTAATTGGCCGGAYTGGGYDTAAAGNG
v6_BC028	V6	TTCTCTACCGG	CCATCTCATCCCTGCGTGTCTCCGACTCAGTTCTCTACCGGAAACTCAAAKGAATTGACGG
v4_BC028	V4	TTCTCTACCGG	CCATCTCATCCCTGCGTGTCTCCGACTCAGTTCTCTACCGGAYTGGGYDTAAAGNG
v6_BC029	V6	TTACCGAAGCG	CCATCTCATCCCTGCGTGTCTCCGACTCAGTTACCGAAGCGAAACTCAAAKGAATTGACGG
v4_BC029	V4	TTACCGAAGCG	CCATCTCATCCCTGCGTGTCTCCGACTCAGTTACCGAAGCGAYTGGGYDTAAAGNG
v6_BC030	V6	TTAACCAGCCG	CCATCTCATCCCTGCGTGTCTCCGACTCAGTTAACCAGCCGAAACTCAAAKGAATTGACGG
v4_BC030	V4	TTAACCAGCCG	CCATCTCATCCCTGCGTGTCTCCGACTCAGTTAACCAGCCGAYTGGGYDTAAAGNG
v6_BC031	V6	TTAGGACTCCG	CCATCTCATCCCTGCGTGTCTCCGACTCAGTTAGGACTCCGAAACTCAAAKGAATTGACGG
v4_BC031	V4	TTAGGACTCCG	CCATCTCATCCCTGCGTGTCTCCGACTCAGTTAGGACTCCGAYTGGGYDTAAAGNG
v6_BC032	V6	TATTCCGGTCG	CCATCTCATCCCTGCGTGTCTCCGACTCAGTATTCCGGTCGAAACTCAAAKGAATTGACGG
v4_BC032	V4	TATTCCGGTCG	CCATCTCATCCCTGCGTGTCTCCGACTCAGTATTCCGGTCGAYTGGGYDTAAAGNG
v6_BC033	V6	TTAACGCCAGG	CCATCTCATCCCTGCGTGTCTCCGACTCAGTTAACGCCAGGAAACTCAAAKGAATTGACGG
v4_BC033	V4	TTAACGCCAGG	CCATCTCATCCCTGCGTGTCTCCGACTCAGTTAACGCCAGGAYTGGGYDTAAAGNG
v6_BC034	V6	TCGTCCTTAGG	CCATCTCATCCCTGCGTGTCTCCGACTCAGTCGTCCTTAGGAAACTCAAAKGAATTGACGG
v4_BC034	V4	TCGTCCTTAGG	CCATCTCATCCCTGCGTGTCTCCGACTCAGTCGTCCTTAGGAYTGGGYDTAAAGNG
v6_BC035	V6	TTAAGACGGCG	CCATCTCATCCCTGCGTGTCTCCGACTCAGTTAAGACGGCGAAACTCAAAKGAATTGACGG
v4_BC035	V4	TTAAGACGGCG	CCATCTCATCCCTGCGTGTCTCCGACTCAGTTAAGACGGCGAYTGGGYDTAAAGNG
v6_BC036	V6	TTACTCGACCG	CCATCTCATCCCTGCGTGTCTCCGACTCAGTTACTCGACCGAAACTCAAAKGAATTGACGG
v4_BC036	V4	TTACTCGACCG	CCATCTCATCCCTGCGTGTCTCCGACTCAGTTACTCGACCGAYTGGGYDTAAAGNG
v6_BC037	V6	TTACACCTCCG	CCATCTCATCCCTGCGTGTCTCCGACTCAGTTACACCTCCGAAACTCAAAKGAATTGACGG
v4_BC037	V4	TTACACCTCCG	CCATCTCATCCCTGCGTGTCTCCGACTCAGTTACACCTCCGAYTGGGYDTAAAGNG
v6_BC038	V6	TAACCTCCGAG	CCATCTCATCCCTGCGTGTCTCCGACTCAGTAACCTCCGAGAAACTCAAAKGAATTGACGG
v4_BC038	V4	TAACCTCCGAG	CCATCTCATCCCTGCGTGTCTCCGACTCAGTAACCTCCGAGAYTGGGYDTAAAGNG
v6_BC039	V6	TTAAGCGTCCG	CCATCTCATCCCTGCGTGTCTCCGACTCAGTTAAGCGTCCGAAACTCAAAKGAATTGACGG
v4_BC039	V4	TTAAGCGTCCG	CCATCTCATCCCTGCGTGTCTCCGACTCAGTTAAGCGTCCGAYTGGGYDTAAAGNG
v4_reverse	V6	none	CCTCTCTATGGGCAGTCGGTGATTACNVGGGTATCTAATCC
v6_reverse	V4	none	CCTCTCTATGGGCAGTCGGTGATACGAGCTGACGACARCCATG

Supplementary Table 4: Primers used in qPCR validation of V6-16S sequencing results and BCoAT/DSR levels.

Gene	Primer ID	Sequence	Reference
16S rRNA	UniF	5’-GTGSTGCAYGGYYGTCGTCA-3’	(Fuller *et al.*, 2007)[1]
	UniR	5’-ACGTCRTCCMCNCCTTCCTC-3’	
Butyryl-CoA: acetyl-CoA transferase (BCoAT)	BCoATscrF	5’-GCIGAICATTTCACITGGAAYWSITGGCAYATG-3’	(Louis and Flint, 2007)[2]
	BCoATscrR	5’-CCTGCCTTTGCAATRTCIACRAANGC-3’	
16S rRNA	16SUNIV-F	5’-GGTGAATACGTTCCCGG-3’	(Kostic *et al.*, 2013)[3]
	16SUNIV-R	5’-TACGGCTACCTTGTTACGACTT-3’	
16S rRNA	Aparv-711F	5'- GGGGAGTATTTCTTCCGTGCCG -3'	This Study
	Aparv-881R	5'- CTTCACCTAAATGTCAAGCCCTGG -3'	
Dissimilatory Sulfite Reductase (DSR)	DSR1-F	5’-ACSCACTGGAAGCACGGCGG-3’	(Bourne *et al.*, 2011)[4]
	DSR1-R	5’-GTGGMRCCGTGCAKRTTGG-3’	

Supplementary Table 5: Primers used in qRT-PCR.

Gene	Primer name	Sequence	Reference
hGAPDH	hGAPDHF	5’-AGAAGGCTGGGGCTCATTTG-3’	(Zhang et al., 2012)[5]
	hGAPDHR	5’-AGGGGCCATCCACAGTCTTC-3’	
TST	hTSTF	5’-TCTCAAGGGCGGTTCCTGGG-3’	This study
	hTSTR	5’-CGTACACGGCCACATCAGGC-3’	
SQRDL	SQRDL951F	5’-AATGCCATCTTCACCTTCCC-3’	This study
	SQRDL1105R	5’-TTAACCCCGAAAATGGCTCC-3’	
COX4-1	hCOX497F	5’-GTGGGCGGTGCCATGTTCTT-3’	This study
	hCOX757R	5’-GCATGGAGTTGCATGGCGGT-3’	
β-actin	β-actinF	5’-TGGAATCCTGTGGCATCCATGAAAC-3’	This study
	β-actinR	5’-TAAAACGCAGCTCAGTAACAGTCCG-3’	
cxcl1	cxcl1F	5’-GCTGGGATTCACCTCAAGAA-3’	This study
	cxcl1R	5’-TCTCCGTTACTTGGGGACAC-3’	
tnf	tnfF	5’-ATGAGCACAGAAAGCATGATC-3’	This study
	tnfR	5’-TACAGGCTTGTCACTCGAATT-3’	
il12p40	il12p40F	5’-GGAAGCACGGCAGCAGCAGAATA-3’	This study
	il12p40R	5’-AACTTGAGGGAGAAGTAGGAATGG-3’	
il-1β	il1bF	5’-GCCCATCCTCTGTGACTCAT-3’	This study
	il1bR	5’-AGGCCACAGGTATTTTGTCG-3’	
il-17a	il17aF	5’-TCCAGAAGGCCCTCAGACTA-3’	This study
	il17aR	5’-ACACCCACCAGCATCTTCTC-3’	

Supplementary Information References

1. Fuller, Z., *et al.* Influence of cabbage processing methods and prebiotic manipulation of colonic microflora on glucosinolate breakdown in man. *The British journal of nutrition* **98**, 364-372 (2007).
2. Louis, P. & Flint, H.J. Development of a semiquantitative degenerate real-time pcr-based assay for estimation of numbers of butyryl-coenzyme A (CoA) CoA transferase genes in complex bacterial samples. *Appl Environ Microbiol* **73**, 2009-2012 (2007).
3. Kostic, A.D., *et al. Fusobacterium nucleatum* potentiates intestinal tumorigenesis and modulates the tumor-immune microenvironment. *Cell Host Microbe* **14**, 207-215 (2013).
4. Bourne, D.G., Muirhead, A. & Sato, Y. Changes in sulfate-reducing bacterial populations during the onset of black band disease. *ISME J* **5**, 559-564 (2011).
5. Zhang, P.J., *et al.* Genes expression profiling of peripheral blood cells of patients with hepatocellular carcinoma. *Cell biology international* **36**, 803-809 (2012).
